# Radiotherapy-Induced Digestive Injury: Diagnosis, Treatment and Mechanisms

**DOI:** 10.3389/fonc.2021.757973

**Published:** 2021-11-05

**Authors:** Guangxia Chen, Yi Han, Haihan Zhang, Wenling Tu, Shuyu Zhang

**Affiliations:** ^1^ Department of Gastroenterology, The First People’s Hospital of Xuzhou, Xuzhou Municipal Hospital Affiliated to Xuzhou Medical University, Xuzhou, China; ^2^ The Second Affiliated Hospital of Chengdu Medical College, China National Nuclear Corporation 416 Hospital, Chengdu, China; ^3^ West China Second University Hospital, Sichuan University, Chengdu, China

**Keywords:** ionizing radiation, radiation-induced digestive injury, gut microbiota, gland transfer, apoptosis, ferroptosis, natural herb, radionuclide-labeled targeting molecule

## Abstract

Radiotherapy is one of the main therapeutic methods for treating cancer. The digestive system consists of the gastrointestinal tract and the accessory organs of digestion (the tongue, salivary glands, pancreas, liver and gallbladder). The digestive system is easily impaired during radiotherapy, especially in thoracic and abdominal radiotherapy. In this review, we introduce the physical classification, basic pathogenesis, clinical characteristics, predictive/diagnostic factors, and possible treatment targets of radiotherapy-induced digestive injury. Radiotherapy-induced digestive injury complies with the dose-volume effect and has a radiation-based organ correlation. Computed tomography (CT), MRI (magnetic resonance imaging), ultrasound (US) and endoscopy can help diagnose and evaluate the radiation-induced lesion level. The latest treatment approaches include improvement in radiotherapy (such as shielding, hydrogel spacers and dose distribution), stem cell transplantation and drug administration. Gut microbiota modulation may become a novel approach to relieving radiogenic gastrointestinal syndrome. Finally, we summarized the possible mechanisms involved in treatment, but they remain varied. Radionuclide-labeled targeting molecules (RLTMs) are promising for more precise radiotherapy. These advances contribute to our understanding of the assessment and treatment of radiation-induced digestive injury.

## 1 Introduction

Cancer is one of the greatest health problems in the 21st century. Approximately 29.8% of premature deaths (4.5 billion out of 15.2 billion) are attributed to cancer, ranking first or second in 134 of 183 countries (1). Radiotherapy, along with chemotherapy and surgery, is one of the three core methods of treating cancer. Nearly 50% of cancer patients receive radiotherapy (2). Compared with surgery, radiotherapy kills target tumor cells with less injury and is preferred when the target tumor tissue/organ cannot be removed. Compared with chemotherapy, radiotherapy limits the involved area and reduces lesions when the tumor is localized. However, radiotherapy is a double-edged sword. That is, even though radiotherapy deals with tumor cells as planned, it may inevitably harm healthy cells.

The digestive system consists of the gastrointestinal tract and the accessory organs of digestion (the tongue, salivary glands, pancreas, liver and gallbladder). During eating, food is chewed by the oral cavity into small pieces and mixed with saliva, forming a bolus that passes through the esophagus into the stomach. Then, the stomach functions to store the food by receptive relaxation. In the stomach, gastric acid and pepsin are secreted, which, aided by the grinding of the stomach wall, turn food into chyme, helping in primary digestion until gastric emptying. Gastric emptying is regulated mainly by inhibitory feedback signals from the duodenum, including both enterogastric inhibitory nervous feedback reflexes and hormonal feedback by cholecystokinin, as well as partly by stomach factors (such as the degree of filling in the stomach and the excitatory effect of gastrin on stomach peristalsis). After the chyme passes into the small intestine, the pancreas secretes various digestive enzymes through the pancreatic bile tract, while the gallbladder releases bile secreted by the liver that breaks down nutrients into molecules to be absorbed in the small intestine. The length of the small intestine, as long as 10 to 16 feet, is helpful for fully absorbing carbohydrates, protein, fat and other nutrients. Then, indigestible food residue passes through the ileocecal valve into the large intestine and forms feces after dehydration. Defecation occurs as a result of reflex contraction of the rectum and relaxation of the anal sphincters (3).

Radiation-induced digestive injury is defined as acute or chronic lesions caused by ionizing radiation in the digestive organs, including the oral cavity, salivary glands, esophagus, stomach, intestines and anus. Radiotherapy, as one of the main methods of cancer treatment, accounts for almost all digestive injuries (4). The digestive system, as one of the most sensitive physiological organs to radiation therapy, usually suffers the most severe side effects from radiotherapy (4).

## 2 Physical Classification of Ionizing Radiation in Radiotherapy

**Figure 1:**
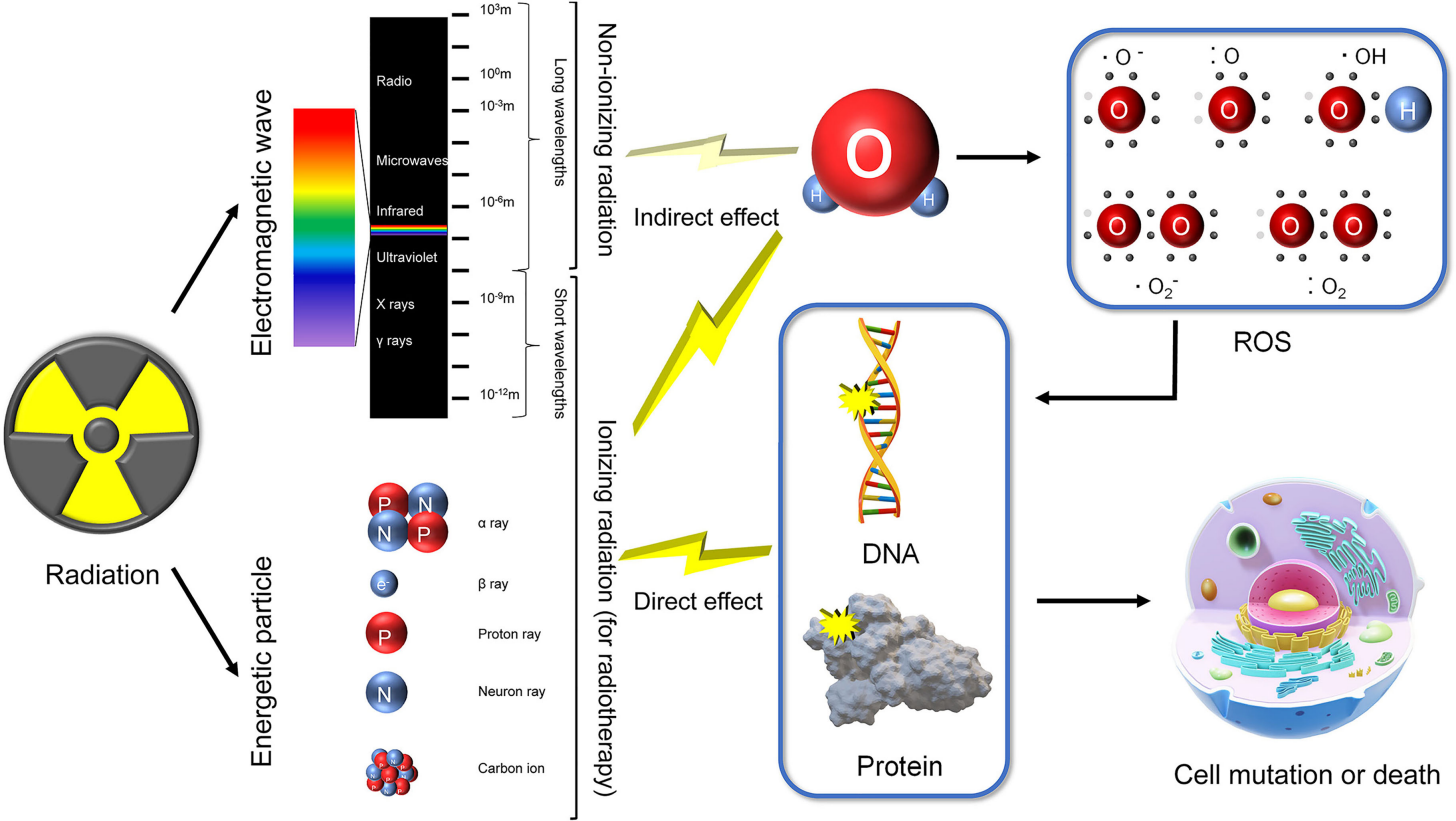
Classification of radiation and mechanisms of radiation-induced injury. Radiation comprises of energetic particles and electromagnetic waves. Energetic particles and short wavelength electromagnetic waves (X rays and γ rays) are classified as ionizing radiation. Longer wavelength electromagnetic waves (>100 nm) are categorized as nonionizing radiation. Ionizing radiation has enough energy to directly break DNA and protein. In addition, ionizing radiation can produce ROS (mainly by ionizing H_2_O), indirectly inducing DNA and protein damage. Nonionizing radiation may also produce little ROS. Impaired DNA and protein finally lead to cell mutation or death.

Not all radiation can be applied to radiotherapy. Ionizing radiation refers to radiation carrying enough energy to ionize atoms and molecules and break chemical bonds. In a broad sense, ionizing radiation varies among different subjects. However, in biology, ionizing radiation is normally defined by the ionization energy of water, the main component of organisms. Nonionizing radiation refers to longer wavelength light including ultraviolet light, visible light, infrared light, microwaves and radiowaves, that cannot break bonds but can cause vibrations characterized as the heat effect. The specific numerical value of ionizing radiation’s energy level is undefined but is usually approximately 12.4 eVs (corresponding wavelength of approximately 100 nm). Ionizing radiation can directly break bonds in DNA and protein. The shorter the wavelengths are, the higher the energy and corresponding radiation-induced damage. This is also true for energetic particles and magnetic waves (X-rays and γ-rays). Energetic particles can be produced by unstable nuclei or by particle accelerators, usually including α-rays (helium), β-rays (electrons), proton rays, neuron rays and heavy ions (**Figure 1**). These energetic particles have strong ionizing effects due to their relatively higher volume and/or charge.

## 3 Pathological Basis for Radiation-Induced Digestive Injury

DNA, proteins and lipids are the basis of cell survival. Their function relies on fine-tuned structure, meaning that there is a high risk of inactivation. Radiation may damage organisms as a result of direct effects, indirect effects and bystander effects. The direct effects refer to the collision of ionizing radiation causing destruction of DNA and/or protein structure, disturbing their functions (5). For indirect effects, both ionizing and nonionizing radiation produce free radicals and reactive oxygen species (ROS). However, compared with ionizing radiation, nonionizing radiation produces much less ROS *via* the heat effect. These highly active products subsequently react with DNA and proteins. The corresponding DNA damage includes single strand breaks, base damage, abasic sites, double strand breaks, non-double strand break clustered lesions, and complex double strand break, some of which are induced by DNA related protein (such as histone) damage (6). Radiation-induced RNA damage manifests as interference in transcription and accelerating in degradation. Both direct and indirect effects finally induce altered gene expression, protein modification, cell death/senescence, and genomic instability (7) (**Figure 1**). The bystander effect is defined from a different perspective. Regarding bystander effects, nonirradiated cells manifest biological changes resulting from transmitted signals from irradiated bystander cells, causing toxic radiation effects on adjacent nonirradiated tissues, usually genomic instability and chromosomal rearrangement (8). Originally, the effects of irradiated bystander cells are derived from direct effects and indirect effects. Both direct effects and indirect effects can function simultaneously, along with bystander effects, working together to induce radiation injury.

Radiotherapy utilizes various types of radiation rays. Each type of radiation ray has advantages and limitations. Compared with traditional photon radiotherapy, including X-rays and γ-rays, protons and heavy ions have much longer wavelengths. As a result, the corresponding diffraction distances are on the same order of magnitude as the tissue size. Radiation diffraction converges on a peak named the Bragg peak (9). By refined calculation, the release of charged particle energy can be limited to the Bragg peak targeting tumor tissue, dramatically reducing the diffusion of radiation (10). Heavy ion therapy has an even narrower Bragg peak than proton therapy, making it more effective against cancer (11). Additionally, heavy ion-radiated tissue manifests as clustered DNA double-strand breaks, enhancing therapeutic efficacy (12). However, protons and heavy ions have larger borders due to their longer wavelengths, making them difficult to locate (13). Comparatively, proton therapy and heavy ion therapy are superior to photon radiotherapy. Unfortunately, proton therapy and heavy ion therapy are severely limited due to their high cost (14). Future improvements in radiation methods for heavy ion therapy may further impel clinical application (15).

## 4 Diagnosis of Radiation-Induced Digestive Injuries

### 4.1 Overall Evaluation

#### 4.1.1 Clinical Features

**Figure 2:**
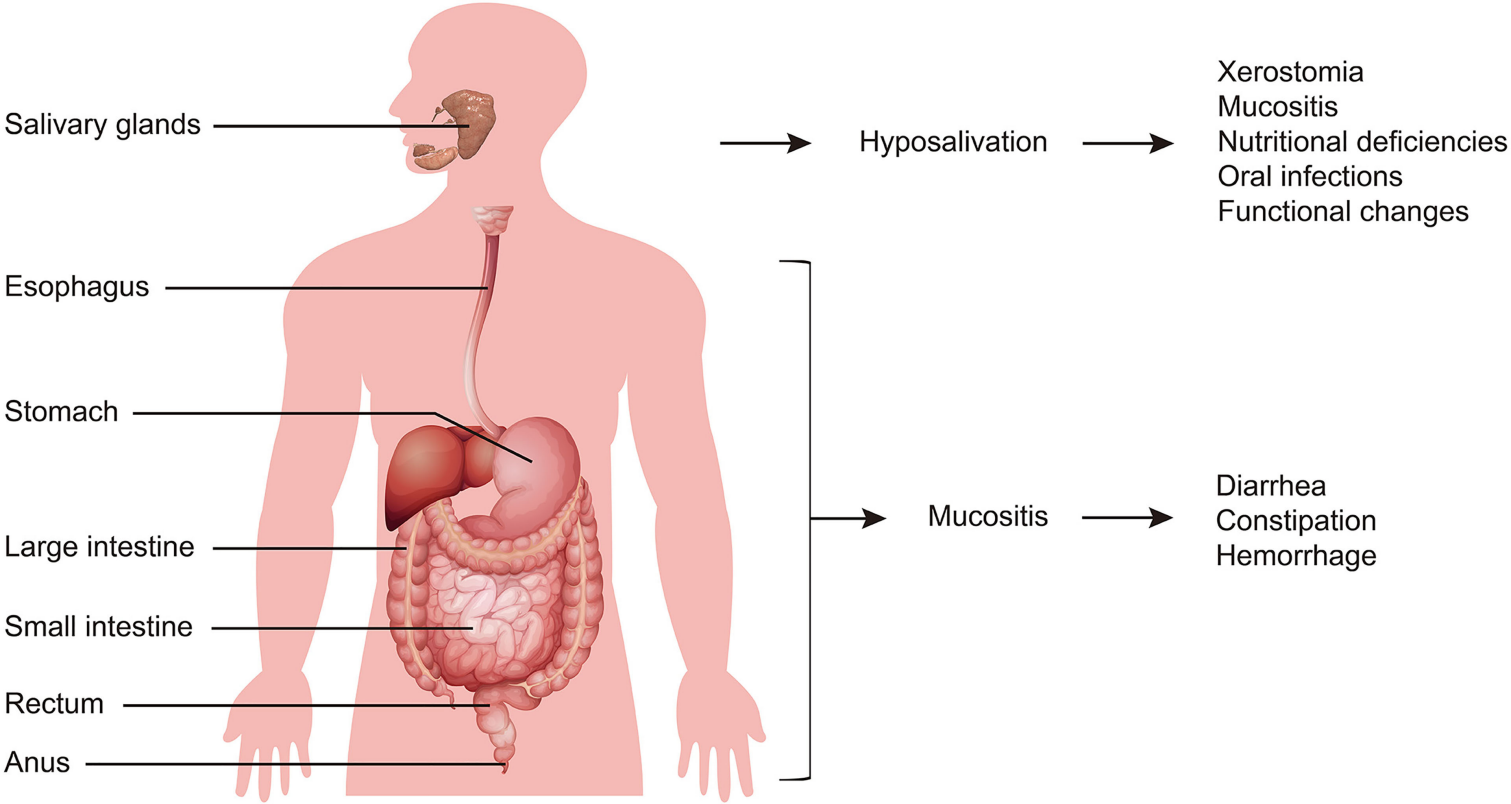
General symptoms of radiation-induced digestive injury. Salivary gland injury is initiated from hyposalivation, and is followed by xerostomia, mucositis, nutritional deficiencies, oral infections, and functional changes. Digestive tract injury starts with mucous inflammation, and then exhibits diarrhea, constipation, and hemorrhage.

The clinical characteristics of radiation-induced digestive injury are summarized in **Figure 2**. Salivary gland injury after radiation directly triggers hyposalivation. Subsequently, a lack of saliva induces xerostomia, mucositis, nutritional deficiencies, oral infections, and functional changes (such as difficulties with mastication, dysphagia and loss of taste) (16, 17). In other digestive tract regions, including the esophagus, stomach, intestine and anus, radiation injury starts with mucous inflammation and is followed by diarrhea, constipation, and hemorrhage (4).

#### 4.1.2 Assessment: Localized Radiation-Induced Digestive Injury

**a) Organ Correlation**

Due to the need for precision medicine as well as reduced side effects, radiotherapy requires that the radiation be confined to the target area. Many studies have proven the efficacy of restricted radiation areas on reduced gastrointestinal side effects as well as enhanced dose tolerance in radiotherapy (18). Usually, radiation injury-related digestive system organs correlate with the surrounding radiotherapy. For example, anal radiotherapy and pancreas radiotherapy correlate with gastrointestinal side effects (19, 20). Radiation of head and neck cancer induces dysphagia (21). Cervical cancer induces sigmoid stricture (22). Generally, periradiotherapy organs can help us locate the possible involved organs. Dose evaluation may help further reduce radiation-induced injury risks.

**b) Dose-Volume Effect**

Radiation-induced digestive injury manifests as a dose-volume effect, meaning that the extent of the lesion highly depends on the radiation dose and radiated volume (23). This theory has been verified in many studies in different organs, including the esophagus (21, 24), stomach (25, 26), small bowel (26, 27), rectum (28–31), and anus. On the basis of the dose-volume effect, radiotherapy-induced injury can be assessed by radiation dose and/or volume calculations. In this way, rectal toxicity (30, 31), acute gastrointestinal toxicity (32, 33), anal toxicity, and salivary gland injury were reported (34–43) and precisely predicted (44). Conversely, Kim et al. found that a higher dose was not associated with cervical esophageal cancer radiotherapy-induced stenosis (45). This conclusion contradicts another study in nasopharyngeal carcinoma patients (46), probably because of the different tumor origins. Which symptoms correlate with dose and/or volume remains unknown. Clinical application lacks a detailed dose-volume standard assessing the radiation-induced risk of each complication. Systematic clinical evidence is necessary for evaluation guidance.

## 5 Imaging Diagnosis

### 5.1 Computerized Tomography (CT)

**Figure 3:**
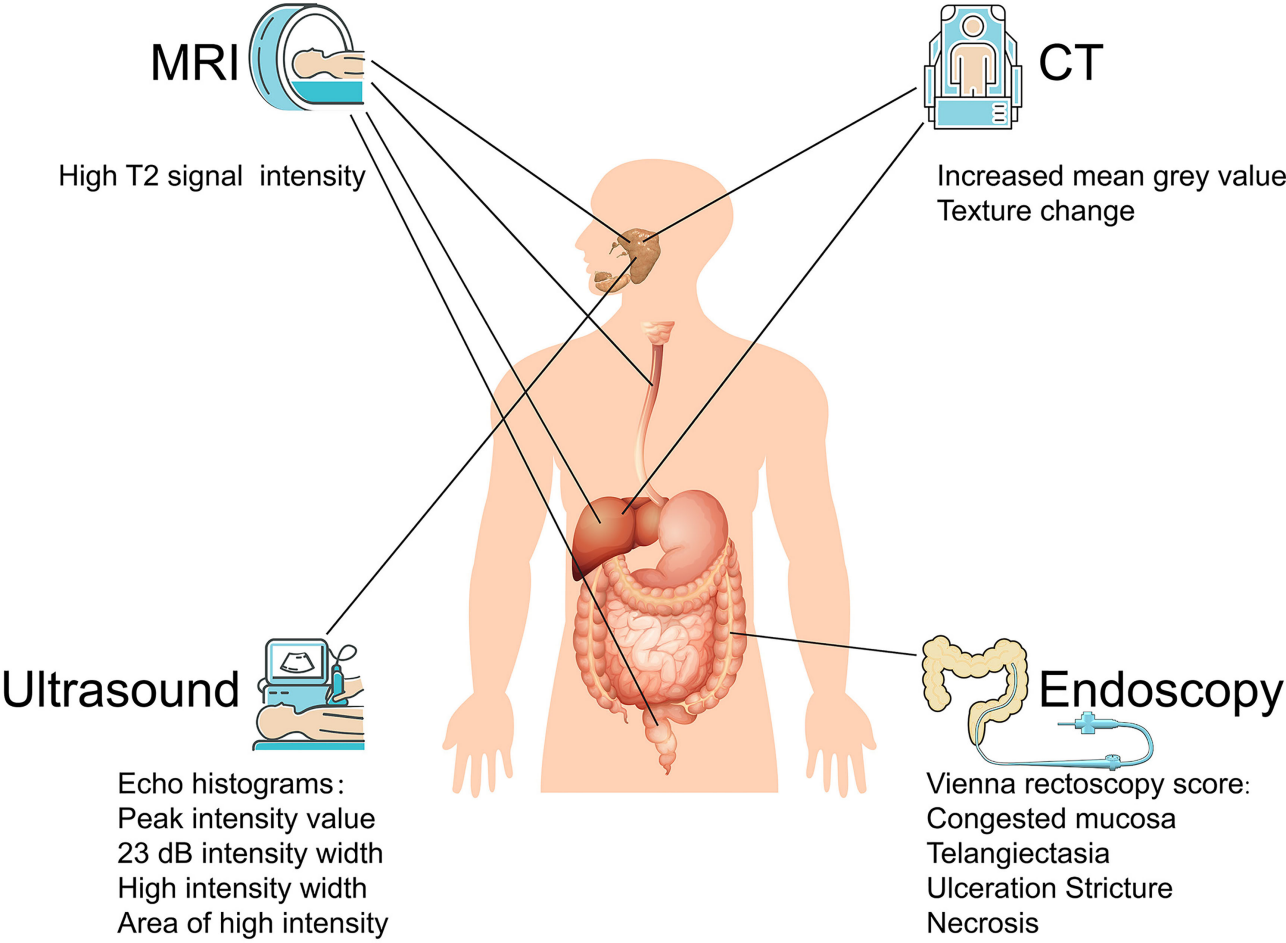
Imaging based diagnosis of radiation-induced digestive injury. For radiation-induced digestive injury, MRI images manifest as high T2 signal intensity; CT images present increased mean grey value and texture change; ultrasound histogram images exhibit shift in peak intensity value, 23 dB intensity width and high intensity width/area; endoscopy discovers congested mucosa, telangiectasia, ulceration, stricture, and necrosis.

CT provides a unique form of cross-sectional imaging. Three-dimensional structures of “slices” of human tissue can be visualized, making CT an effective approach to predict radiation-induced injury. CT textural features could be used in combination with volume to characterize structural modifications of the parotid glands and to predict parotid shrinkage at the end of radiotherapy (47). By nonenhanced CT, a reduction in the volume of the parotid and submandibular glands and an increase in attenuation of the parotid gland can help grade radiation-induced salivary dysfunction (48). Parotid gland CT volume and density during head and neck cancer can also predict acute xerostomia (49). In summary, CT images of radiation-induced salivary injury are characterized by an increased mean gray value or density in the early stage, followed by shrinkage of the glands; texture analysis of CT is another indicator for assessing radiation-induced acute xerostomia (50) (**Figure 3**). Moreover, ^18^F-FDG PET image biomarkers have considerably improved the prediction of late radiation-induced xerostomia (51), which is a promising method. Liver injury usually appears as CT imaging changes, and cases of CT assessing radiation-induced liver injury have been reported (52), suggesting that CT may help in the evaluation of radiation-induced liver injury. Although changes in CT images can be observed during radiotherapy, the variation in the liver is too small to diagnose, limiting CT to only prepared assessments that are started before radiation (53). Additional technologies may improve the CT diagnostic rate. For instance, single-photon emission CT imaging of mice precisely diagnosed radiation-induced liver disease (54). The diagnosis of other digestive organs by CT has rarely been reported.

### 5.2 Ultrasonic Histogram

Ultrasonic elastography, as a new ultrasound diagnostic technique, calculates the strain distribution by echo signals before and after compression and deformation of the tissue to obtain elastic (hardness) characteristic information for efficient clinical diagnosis. However, elastic noise usually interferes with imaging quality. Histogram matching algorithms can help suppress noise signals, accelerating the application of ultrasound histograms in many diseases. The efficacy of ultrasonic histogram analyses has been validated in salivary gland injury. Yang et al. used sonographic features as imaging signatures to assess radiation-induced parotid injury (55). They then summarized a family of sonographic features derived from echo histograms, including the peak intensity value, 23 dB intensity width, high intensity width and area of high intensity (56) (**Figure 3**). In addition, they further concluded that ultrasound histogram features (especially receiver operating characteristic curves) can be used to measure acute and late toxicity of the parotid glands after head and neck cancer radiotherapy, which may be developed into a low-cost imaging method for xerostomia monitoring and assessment (57). Salivary gland dysfunction, which relies on the blood supply, is easy to diagnose by ultrasound histogram. Other digestive organs, which have little external vascular variation compared with their surroundings, plus their deeper location, appear to have no distinguishable ultrasonic and CT distinctions.

### 5.3 Magnetic Resonance Imaging (MRI)

MRI, as a radiation-free medical imaging technique, is gradually replacing CT scans in clinical applications. MRI works by polarization of hydrogen atoms and has proven to be effective in diagnosing radiation-induced salivary gland injury, esophageal injury, liver injury, and rectal injury (58–61). MRI images of radiation injury generally manifest as high signal intensity in T2, pathologically based on tissue edema. For acute radiation injury, an obvious shift in the T2 weighted imaging (T2WI) signal can be observed in the radiated area; for delayed radiation injury, the involved tissue may only exhibit a slight change on T2WI (62) (**Figure 3**).

### 5.4 Endoscopy

Early endoscopic findings deemed the Vienna rectoscopy score useful for predicting the possibility of late clinical radiation proctitis (63). Specific standards include congested mucosa, telangiectasia, ulceration, stricture, and necrosis (**Figure 3**). Radiation-induced enteritidis can be diagnosed by wireless capsule endoscopy (64, 65). Nevertheless, when it develops into obvious endoscopic manifestations, radiation injury is usually accompanied by other diagnostic clinical symptoms. Despite its low sensitivity in diagnosis, endoscopy may help in the prognosis as well as in essential treatment such as hemorrhage. The American Society for Gastrointestinal Endoscopy issued guidelines on the role of endoscopy for bleeding in chronic radiation proctopathy in 2019. These guidelines focused on currently available endoscopic therapies for managing patients with chronic radiation proctopathy, which include argon plasma coagulation, bipolar electrocoagulation, heater probe, radiofrequency ablation, and cryoablation (66). Further studies improving endoscopic standards to diagnose radiation proctopathy may lead to further refinement of these guidelines.

## 6 Nonimaging Diagnosis

### 6.1 Gut Microbiota

The gut microbiota has become a new focus of various diseases, including chronic liver disease (67), type 2 diabetes mellitus (68), inflammatory bowel diseases (69), cardiovascular disease (70), sarcopenia (71) and cancer (72). Its correlation with radiation sensitivity has also been reported (73). A study in mice indicated that conventional intestinal microbiota composition may predict radiation injury (74). The control of bacterial translocation affects gastrointestinal acute radiation syndrome in mice (75). The prediction mechanism may involve pyrimidine and tryptophan pathways (76). Furthermore, a series of metabolic profile data of gut microbiota in cervical cancer patients summarized that radiation-induced acute intestinal symptoms are characterized by increased fecal concentrations of α-ketobutyrate, valine, uracil, tyrosine, trimethylamine N-oxide, phenylalanine, lysine, isoleucine, glutamine, creatinine, creatine, bile acids, aminohippurate, and alanine, accompanied by reduced concentrations of α-glucose, n-butyrate, methylamine, and ethanol (77). This study lays a solid foundation for the diagnosis and prediction of intestinal radioinjury. Analysis of the gut microbiota along with metabolic products is a promising method evaluating the severity of radiation-induced intestinal injury.

### 6.2 Other Predictive Factors

Moreover, some other factors should not be ignored. Substantial gland loss in the anterior rectal walls can predict radiation-induced late clinical proctitis (78). Single nucleotide polymorphisms and copy number variations were also reported to predict radiation rectal toxicity (79). Other metabolic-related nutrients, such as vitamin D (80) and citrulline (81), may serve as markers for radiation injuries. Besides, oral flora may also help diagnose radiation-induced injury, usually characterized by overgrowth of specific fungi such as Candida albicans (82, 83).

## 7 Precaution and Treatment for Radiation-Induced Digestive Injury

### 7.1 Precaution

#### 7.1.1 Gland Transfer

Salivary glands have relatively separate structures and can be isolated for transplantation to avoid radiation injury. This theory has been proven by various studies, especially for head and neck cancer radiotherapy and nasopharyngeal carcinoma-induced xerostomia (84, 85). Moreover, although fails to relieve dysphagia (86), gland transfer does not affect long-term treatment efficacy (85). A phase II study found that the technique of submandibular salivary gland transfer is reproducible in a multicenter setting (87). Further phase III randomized studies proved that submandibular salivary gland transfer is effective in curing radiation-induced xerostomia (88). Similar conclusions were reproduced in a meta-analysis (89). More phase III clinical studies may be required to evaluate the efficacy of gland transfer to promote the clinical application of gland transfer in radiation-induced salivary lesions.

#### 7.1.2 Improvement in Radiotherapy

**a) Shielding**

Shielding of the sensitive part of the target area is a traditional way to avoid radiation-induced injury. For example, partial shielding of the oral cavity in rhesus macaques may prevent oral mucositis (90). However, it is difficult to shield the visceral organs. Hydrogels precisely solve this problem. Hydrogels are three-dimensional cross-linked polymer networks that can absorb and retain large amounts of water, meaning that they are not poisonous to humans. This feature allows hydrogels to easily absorb radiation, similar to normal tissue. Implantation of hydrogel between the target tissue area and radiosensitive normal structure can effectively reduce the radiation volume of the normal structure. As proof, a simulation in cadaveric models of oropharynx cancer treated with intensity-modulated radiation therapy (IMRT) found that the hydrogel reduces the salivary gland radiation dose (91). Reductions in the radiated dose were verified in patients (92). In the clinic, rectum spacer hydrogel implantation prevents rectal injury in prostate cancer radiotherapy (93). Hydrogel spacers decreased duodenum radiation in pancreatic cancer radiotherapy (94). In addition, improvement in gastrointestinal syndrome was reported after prostate radiotherapy (95, 96). Hydrogels have been widely used in clinical practice. Traditional hydrogels are preshaped and are usually implanted *via* operation. Compared with traditional hydrogels, injectable hydrogels have the advantages of eliminating operation limitations and drug administration but have accompanying high risks of inflammation and dislocation (97). Improvement in hydrogels, such as adding anti-inflammatory drug components or using other inflammation-free hydrogels, may avoid inflammation. For instance, *in situ* photo-cross-linking hydrogels can restore the hypoxia-inducible factor 1-alpha pathway (98). Pectin/polyacrylamide hydrogels successfully deliver budesonide to the colon (99). Tannic acid acts as a cross linker and additionally enhances the anti-inflammatory properties of hydrogels (100). Topical hydrogels containing *Achyrocline satureioides* oily extract can reduce inflammation (101). Dexamethasone-loaded thermosensitive hydrogels suppress inflammation in rheumatoid arthritis (102). All of these findings suggest promising application of improved injectable hydrogels in radiotherapy.

**b) Dose Distribution**

The dose distribution of radiotherapy influences radiation-induced injuries. High-dose-rate monotherapy can relieve radiation toxicity compared with low-dose-rate multitherapy (103). High-dose-rate boost treatment is associated with fewer side effects (104). Traditional radiotherapy is limited by dose administration to avoid radiotoxicity to normal tissues. Fractioned radiotherapy increases total dose tolerance and reduces the number of visits and the total cost of treatment without increasing radiotoxicity (105). In contrast, hypotreated prostate cancer patients suffered from significantly increased late genitourinary toxicity (106). In contrast, in the latest studies comparing hyperfractionated radiotherapy, conventionally fractionated radiotherapy, and hypofractionated radiotherapy, although relatively lower-fractionated radiotherapy may increase acute toxicity, there appears to be no significant difference in the long-term effects or late toxicity (105, 107–112). More systematic studies are required to determine whether fractionated radiotherapy is superior to conventional radiotherapy.

### 7.2 Treatment

#### 7.2.1 Mesenchymal Stem Cells (MSCs)

MSCs are widely defined as a plastic-adherent cell population that can be directed to differentiate *in vitro* into osteogenic, chondrogenic, adipogenic, myogenic, and other lineages. MSC differentiation potential is widely used in tissue repair. MSCs have been proven to be able to restore radiation-induced injury (113, 114). For example, adipose-derived stromal cells have the potential to restore salivary gland function after irradiation, as evidenced by the restoration of blood flow within submandibular gland tissue (115). Furthermore, human adipose tissue-derived stem cells alleviate radiation-induced xerostomia (116). Salivary gland stem cells can also ameliorate radiation-induced hyposalivation (117). Stem cell transplantation not only rescues hyposalivation but also restores tissue homeostasis in the irradiated gland, which is necessary for long-term maintenance of adult tissue (118). Administration of adipose-derived stem cells immediately after radiation at a dose of 18 Gy can protect both the morphology and function of the salivary glands eight weeks after radiation in mice (119). In summary, MSCs can ameliorate radiation-induced salivary injury, including xerostomia (120, 121).

Compared with radiation-induced salivary injury, the efficacy of MSCs in other digestive organs remains variable. Related research is summarized as follows: in a rat model of radiation-induced esophageal injury, dental pulp stem cell transplantation exhibited a therapeutic effect (122). For the colorectum, one study showed that MSCs may reverse radiation injury (123). Autologous bone marrow-derived mesenchymal stem cells may improve radiation-induced proctitis (124). Adipose-derived stem cells may facilitate the repair of defects in maxillofacial soft tissue (125). These cases alone hardly prove the viewpoint. Nonetheless, these results suggest that MSCs may have therapeutic potential for radiotherapy-induced tissue damage (126). Unfortunately, the specific mechanisms of MSC-based treatment have rarely been investigated among the studies, except that platelet-rich plasma improves the therapeutic efficacy of MSCs (127).

Chang et al. investigated the therapeutic mechanisms of MSCs and found that human adipose-derived mesenchymal stem cells (hAd-MSCs) had postradiation healing effects, including anti-inflammation, neovascularization and maintenance of epithelium homeostasis, as indicated by the elevated serum IL-10, upregulation of vascular endothelial growth factor, basic fibroblast growth factor and epidermal growth factor in irradiated intestine, mobilization of CD31-positive hematopoietic stem cells or hematopoietic progenitor cells, and the prolonged presence of Bmi1-positive cells within crypts. The authors found that irradiated rats survived longer than nontreated animals (128). More related research is warranted in further studies.

#### 7.2.2 Bone Marrow Transplantation

Bone marrow, similar to digestive system organs, is often involved in radiation-induced injury. Transplantation of bone marrow is a traditional way to cure bone marrow lesions. Improvement in bone marrow transplantation not only restores hematopoietic function but also alleviates other digestive symptoms (129, 130). Bone marrow-derived cells can also reduce radiogenic oral mucositis (131). To further determine how bone marrow restores digestive symptoms, Tran et al. injected bone marrow soluble extract (“soup”) into mice and found that bone marrow soup restored salivary flow rates to normal levels; protected salivary acinar, ductal, myoepithelial, and progenitor cells; increased cell proliferation and blood vessels; and upregulated the expression of tissue remodeling/repair/regenerative genes. Bone marrow soup can be advantageously used to repair irradiation-damaged salivary glands rather than transplanting whole live bone marrow cells which carry the risk of differentiating into unwanted/tumorigenic cell types in the salivary glands (132). Further study suggests that bone marrow transplantation recruits host myelomonocytic cells and enhances intestinal stroma proliferation after radiation by secreting cytokines that enhance angiogenesis and chemotaxis (133). Bone marrow transplantation may share common mechanisms with MSCs in radiation-induced injury restoration. Controlled studies of MSCs and bone marrow transplantation may reveal interesting mechanisms.

#### 7.2.3 Gut Microbiota

Since the gut microbiota can predict radiation injury, it is quite likely that modulation of the gut microbiota could minimize radiation injury. The gut microbiota plays a major role in the pathogenesis of radioinjury through the modification of intestinal barrier function, innate immunity and intestinal repair mechanisms (134). We determined the correlation between gut microbiota, metabolites, and radiation injury in **Table 1** (135–139).

**Table 1:** Metabolic products and possible sources related to radiation-induced injury.

Subjects	Dose	Metabolic products	Sources	Effects	Reference
C57BL/6 mice (male & female)	9.2 Gy	Propionate and tryptophan	*Lachnospiraceae* and *Enterococcaceae*	Alleviate acute radiation syndrome	(133)
C57BL/6 mice (male & female)	21 Gy	Butyrate	Butyrate-producing bacteria	Reduce cell radiosensitivity	(134)
C57BL/6 mice (male & female)	12 Gy	Indole 3-propionic acid	Tryptophan related gut microbiota product	Alleviate acute radiation syndrome	(135)
C57BL/6 mice (male)	9 Gy	Urolithin A	Metabolite of ellagitannin	Alleviate ionizing radiation-induced intestinal damage	(136)
C57BL/6 mice (male)	15 Gy	Phosphatidylcholines (36:0e)	*Alistipes*	Related to radiation enteritis	(137)
C57BL/6 mice (male)	15 Gy	Diglyceride (18:0/20:4)	*Bacteroides*	Related to radiation enteritis	(137)
C57BL/6 mice (male)	15 Gy	Phosphatidylcholines (35:2)	*Dubosiella*	Related to radiation enteritis	(137)
C57BL/6 mice (male)	15 Gy	Phosphatidylcholines (35:6)	*Eggerthellaceae*	Related to radiation enteritis	(137)
C57BL/6 mice (male)	15 Gy	Triglyceride (18:2/18:2/20:4)	*Escherichia-Shigella*	Related to radiation enteritis	(137)

Characteristic changes in the structure of the gut microbiota after radiation (such as *Bacteroides*) can serve to predict radiation injury (140). Meanwhile, interference of gut microbiota may lessen radiation toxicity (141). Measures regulating gut microbiota include probiotics (142), a methionine diet (143), hydrogen-water oral gavage (144), and omega-3 polyunsaturated fatty acids (ω–3 PUFAs) (145). Cui et al. reported the sex related effects for gut microbiota in relieving radiation injury (146). Notably, a large proportion of therapeutic drugs for radiation induced injury have effects on estrogen receptors and downstream effectors. This finding highlighted the importance of sex related receptors in treating radiation-induced injury. Nonetheless, these are all animal model studies with low reliability. Recently, Guo et al. transferred human and mouse radiation survivors’ gut microbiota by fecal engraftment and dirty cage sharing and found improved radiation-induced injury related to *Lachnospiraceae* and *Enterococcaceae*. Two tryptophan pathway metabolites of these two bacteria, namely, 1H-indole-3-carboxaldehyde and kynurenic acid, provided long-term radioprotection. This is the first study proving the efficacy of gut microbiota modulation in humans, laying a foundation for clinical intervention of the human gut microbiota against radiation injury. All these cases prove that the gut microbiota presents opportunities to predict, prevent, and treat radiation lesions (147). Future targeting of patient-tailored restoration of optimal microbial composition could lead to a new era of radioprotection (148).

#### 7.2.4 Related Therapeutic Drugs and Possible Mechanisms

The reported radioprotective agents are divided into several categories: free radical scavengers [such as thiols and amines (esp. aminothiols and phosphorothioates)], redox stabilizers (such as superoxide dismutase), antioxidant nutrients (vitamin A, B, C, E, and their related metabolites or analogues (such as β-carotene and folic acids), selenium derivatives, and phytochemicals (149). The overall effects of these drugs have been verified. With the development of modern biotechnology, many new drugs have proved their effectiveness in radiation-induced injury. We summarize representative mechanisms as well as updated drugs below.

**a) Cell Death in Radiation-Induced Digestive Injury**

Radiation-induced digestive injury induces cellular responses. These responses have mutual effects, and it is difficult to determine the dominant pathways. Cell autophagy, cell cycle arrest and even cell death have been reported in response to radiation (150–188). Here, we focused on cell death related pathways, especially apoptosis and ferroptosis in radiation-induced digestive injury.

**b) Apoptosis in Radiation-Induced Digestive Injury**

**Figure 4:**
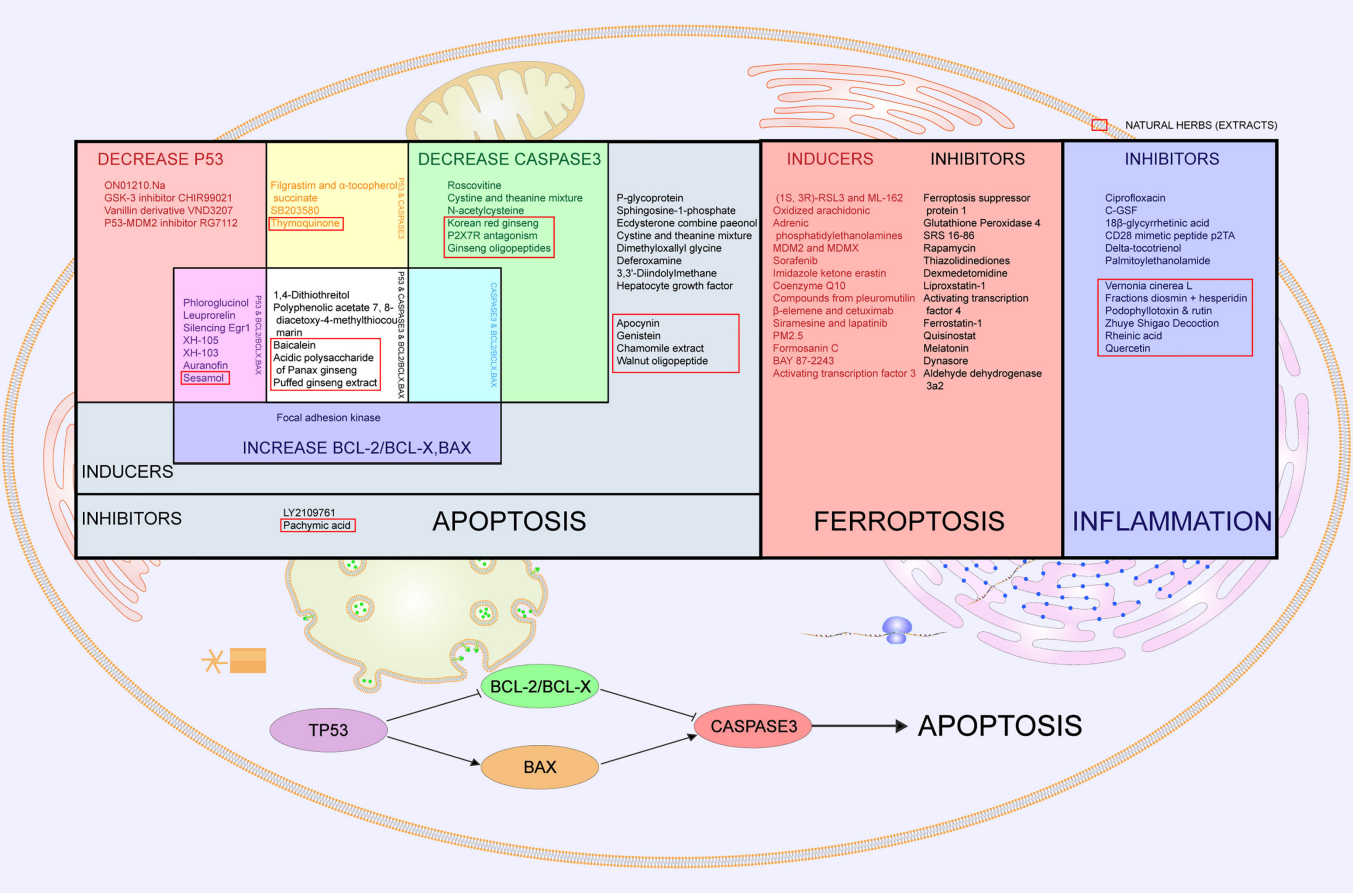
Cell death- and inflammation-related drugs and countermeasures in radiation-induced digestive injury. Cell death-related drugs and countermeasures are divided into apoptosis and ferroptosis. In the left part of the table labeled “Apoptosis”, all listed drugs prove to be effective in radiation-induced digestive injury. Grey part represents apoptosis inhibitors and inducers with no specific targets. The red part represents drugs that decrease p53, and the green part means drugs that decrease caspase3. Blue is for drugs that increase bcl-2/bcl-x or bax. Overlapping parts for two of the above three kinds of drugs are painted magenta, yellow and cyan respectively, meaning that drugs regulate both two factors. White stands for drugs with all of three functions. Clustering of drugs regulating p53, bcl-2/bcl-x, bax and caspase 3 implies that the p53 pathway is activated. The middle square painted red lists inducers and inhibitors of ferroptosis. The right square in blue lists inhibitors of inflammation that alleviate radiation-induced digestive injury. Natural herbs are selected with red boxes.

Multiple studies have reported the anti-radiation effectiveness of apoptosis-related drugs such as genistein (161), P-glycoprotein (163), sphingosine-1-phosphate (162), ecdysterone combined with paeonol (164), cystine and theanine mixture (153), apocynin (165), dimethyloxallyl glycine (166), deferoxamine (167), 3,3’-diindolylmethane (168), hepatocyte growth factor (169), and walnut oligopeptide (170) (**Figure 4**), indicating that regulating apoptosis may alleviate radiation injury (160). Apoptosis-promoting drugs such as LY2109761 (TGF-β receptor inhibitor) (171) and pachymic acid (172) may act as radiotherapy sensitizers, subsequently allowing for a reduction in the radiation dose and normal tissue injury.

Among the anti-radiation drugs that act *via* apoptosis, TP53 (p53) is most frequently involved. TP53 is the most easily compromised gene target modulating cell behavior (189) and participates in radiation-induced digestive injury. p53 is involved in many pathways, including p38/p53/p21 (senescence related) (190), p53/Reprimo (cell cycle arrest at G2/M) (191), Gadd45/p38/p53 (cell cycle checkpoints, apoptosis, and DNA repair), p53-FAS (apoptosis receptor in cell membrane) (192), PIDD (P53-induced protein with a death domain) (193), p53/bcl-2/Bax (apoptosis pathway) (194), p53-inducible genes (195), p53/Scotin (cell cycle arrest, apoptosis) (196), and ATF6/p53/AIFM2 (197).

Caspase 3 also participates in the apoptosis pathway (163). Caspase 3-related drugs include roscovitine (150), SB203580 (151), filgrastim and α-tocopherol (152), cystine and theanine mixtures (153), acidic polysaccharides of *Panax ginseng* (154), Korean red ginseng (155), P2X7R antagonism (156), ginseng oligopeptides (157), thymoquinone (158), and N-acetylcysteine (159) (**Figure 4**).

Since p53 plays various roles in radiation-induced injury, it is unclear which effect is dominant. Coincidently, the summarized related drugs that attenuate radiation injury present clustering of p53, bcl-2/bcl-x, bax and caspase 3. Among antiapoptotic changes, including decreasing p53, decreasing caspase 3 and increasing bcl-2/bcl-x or bax, most drugs induce more than one effect (**Figure 4**). This discovery strongly supports the p53/bcl-2/bax pathway as dominant in radiation-induced digestive injury (174, 176–179, 181–186, 188) (**Figure 4**). Other p53-related drugs that have curative effects in radiation-induced digestive injury, such as Ex-RAD (^®^) (173), may share the same pathway. Nevertheless, knockout of p53 or p21 paradoxically accelerates gastrointestinal damage and death, indicating that p53 may have a bidirectional effect in radiation-induced injury (198).

**c) Ferroptosis in Radiation-Induced Digestive Injury**

Ferroptosis is an iron-dependent type of programmed cell death initiated by lipid peroxide accumulation and depletion of plasma membrane polyunsaturated fatty acids (199). Traditionally, ferroptosis is regulated by amino acid and glutathione metabolism, lipid metabolism, and iron metabolism (200). Radiotherapy may also induce ferroptosis (201, 202). Specific mechanisms involve promotion of lipid peroxidation, interruption of the scavenging capacity of PUFA-PL-OOH, and activation of peroxisomes (203). Radiation induces the expression of ACSL4, a lipid metabolism enzyme required for ferroptosis, resulting in elevated lipid peroxidation and ferroptosis (204). The DNA damage response is another target that explains ferroptosis after radiotherapy, mainly by affecting the function of GPX4 and FSP1 and their respective cofactors, GSH and CoQ10 (205).

Many studies unanimously confirmed that inhibition of ferroptosis alleviates radiation injury (206–209). For example, evidence shows that AMPK activation may inhibit ferroptosis and thus may help reduce radiation-induced injury (210). Similarly, ferroptosis inhibitors decrease ROS and inflammatory cytokine levels in radiation-induced lung injury (211). Other ferroptosis inhibitors, such as p53, PEBP1, ENPP2, and phospholipase iPLA (2) β, may also serve as radiation protectors (212–229). Ferroptosis inducers have the potential to be effective radiosensitizers for radiotherapy (230–248) (**Figure 4**).

**d) Inflammation in Radiation-Induced Digestive Injury**

Inflammation-related cytokines are another high-frequency group of anti-radiation drugs for digestive injury. IL-6-related anti-radiation drugs include ciprofloxacin (249), C-GSF (250), 18-β-glycyrrhetinic acid (251), CD28 mimetic peptide p2TA (252), delta-tocotrienol (253), and palmitoylethanolamide (254), suggesting that inflammatory inhibitors may also contribute to radiation injury (**Figure 4**). Even so, the American Society for Gastrointestinal Endoscopy (ASGE) guidelines on the role of endoscopy for bleeding from chronic radiation proctopathy recommended not using anti-inflammatory drugs because they lacked clinical evidence (66). The efficacy and safety of anti-inflammatory drugs and countermeasures warrant further investigation.

**e) Natural Herbs (or Extractions) Against Radiation-Induced Digestive Injuries**

In addition to modern synthetic drugs, traditional herbs play an indispensable role in curing radiation-induced digestive injury. Most of these effective herbs have been reported to regulate cell death (mainly apoptosis), including tea polyphenols (255), genistein (161), pachymic acid (172), sesamol (175), baicalein (180), acidic polysaccharide of *Panax ginseng* (154), explosively puffed ginseng (187), and resveratrol (256). Some herbs are involved in inflammation pathways, such as *Vernonia cinerea* L (257), fractions of diosmin + hesperidin (258), podophyllotoxin + rutin (259), Zhuye Shigao decoction (260), and rheinic acid (261) (**Figure 4**). Apocynin protects against radiation-induced injury by reducing apoptosis and oxidative stress-derived inflammation (165). Similarly, chamomile extract and walnut oligopeptides are also involved in both apoptotic and inflammatory pathways (170, 262). Quercetin increases aquaporin 5 expression and calcium uptake, thus suppressing radiation-induced oxidative stress and inflammatory responses (263). Glycyrrhizin protects γ-irradiated mice from gut bacteria-associated infectious complications by improving miR-222-associated Gas5 RNA reduction in macrophages at the bacterial translocation site (264). There are several curative herbs without corresponding mechanisms, with only morphological improvement, including *Lagenaria siceraria* extract (265), triphala (266), and resveratrol (267). Natural herbs are a great source of active compounds for reducing radiation-induced digestive injury. More research investigating the underlying mechanisms may reveal new therapeutic targets.

## 8 Future Perspectives

Radiation-induced digestive injury remains a dominant problem since the application of radiotherapy. The current means of diagnosis and treatment are still far from satisfactory. Specific clinical guidelines supported by valid data are urgently needed. In diagnosis, artificial intelligence and deep learning can integrate comprehensive information including clinical features, imaging manifestations, and other predictive factors. Based on the antigen-antibody reaction and affinity interaction, specific biomarkers can be labeled by radionuclides and specifically targeted in diagnosis and treatment (268). For example, ^89^Zr-labeled anti-γH2AX has successfully shown a radiobiological response in PET-CT (269). It is expected that radionuclide-labeled targeting molecules (RLTMs) may be used to precisely diagnose and evaluate radiation damage. Moreover, according to the biological effect of targeted biomarkers, aided by tissue-specific binding sites, RLTMs may act as radiotherapy sensitizers and radio-protectors. The combined application of RLTMs can provide an all-around assessment and strategies for multifunctional treatment. In precaution, novel regenerative peptide may prevent radiation-induced injury (270). In treatment, stem cell regeneration as well as gut metabolites application has shown their promise ameliorating radiotherapy-induced injury. However, there is still a long way from lab bench to bedside.

## 9 Conclusions

In general, radiation-induced digestive injuries during radiotherapy can be divided into two categories: salivary gland injury and digestive tract injury. For salivary gland injury, radiation damage derives from hyposalivation, followed by xerostomia, mucositis, nutritional deficiencies, oral infections, and functional changes. The unique anatomical structure of the salivary gland makes it easier to diagnose injury in these glands by CT, US, and MRI. Gland transfer is a promising method for preventing radiation damage. For digestive tract injury, the involved organ correlates with the radiated area, and the initial symptom is mucous inflammation, followed by diarrhea, constipation, and hemorrhage. Microbiota modulation may become an effective way of reducing radiation-induced gastrointestinal syndrome. Both salivary gland injury and digestive tract injury can be relieved by shielding, dose redistribution, mesenchymal stem cell transplantation and bone marrow transplantation. Inhibitors of cell death and inflammation may be an effective approach for reducing radiation-induced digestive injury. Natural herbs leave plenty of therapeutic potential to be discovered. We concluded that RLTMs are a promising technique in radiotherapy.

## Author Contributions

GC: Topic Presentation and Review Structure Control. SZ: Review Modification and Improvement. YH: Review Writing. HZ: Illustration design and Review Typeset. WT: Illustration design. All authors contributed to the article and approved the submitted version.

## Funding

This work is supported by the National Natural Science Foundation of China (82073477 and 82003390), the Technology Innovation Project of Chengdu (2021-YF05-01603-SN) and the Young Talent Program of China National Nuclear Corporation.

## Conflict of Interest

The authors declare that the research was conducted in the absence of any commercial or financial relationships that could be construed as a potential conflict of interest.

## Publisher’s Note

All claims expressed in this article are solely those of the authors and do not necessarily represent those of their affiliated organizations, or those of the publisher, the editors and the reviewers. Any product that may be evaluated in this article, or claim that may be made by its manufacturer, is not guaranteed or endorsed by the publisher.

## References

[1] World Cancer Report: Cancer Research for Cancer Prevention. Available at: http://publications.iarc.fr/586.

[2] Araujo IK Muñoz-Guglielmetti D Mollà M. Radiation-Induced Damage in the Lower Gastrointestinal Tract: Clinical Presentation, Diagnostic Tests and Treatment Options. Best Pract Res Clin Gastroenterol (2020) 48-49:101707. doi: 10.1016/j.bpg.2020.101707 33317789

[3] Hall JE. Guyton and Hall Textbook of Medical Physiology. Philadelphia, PA: Elsevier (2016).

[4] Shadad AK Sullivan FJ Martin JD Egan LJ. Gastrointestinal Radiation Injury: Symptoms, Risk Factors and Mechanisms. World J Gastroenterol (2013) 19(2):185–98. doi: 10.3748/wjg.v19.i2.185 PMC3547560 23345941

[5] Wei J Wang B Wang H Meng L Zhao Q Li X. Radiation-Induced Normal Tissue Damage: Oxidative Stress and Epigenetic Mechanisms. Oxid Med Cell Longev (2019) 2019:3010342. doi: 10.1155/2019/3010342 31781332 PMC6875293

[6] Sharma R Lewis S Wlodarski MW. DNA Repair Syndromes and Cancer: Insights Into Genetics and Phenotype Patterns. Front Pediatr (2020) 8:570084. doi: 10.3389/fped.2020.570084 33194896 PMC7644847

[7] Painuli S Kumar N. Prospects in the Development of Natural Radioprotective Therapeutics With Anti-Cancer Properties From the Plants of Uttarakhand Region of India. J Ayurveda Integr Med (2016) 7(1):62–8. doi: 10.1016/j.jaim.2015.09.001 PMC4910298 27240731

[8] Rusin A Seymour C Mothersill C. Chronic Fatigue and Immune Deficiency Syndrome (CFIDS), Cellular Metabolism, and Ionizing Radiation: A Review of Contemporary Scientific Literature and Suggested Directions for Future Research. Int J Radiat Biol (2018) 94(3):212–28. doi: 10.1080/09553002.2018.1422871 29297728

[9] Malouff TD Mahajan A Krishnan S Beltran C Seneviratne DS Trifiletti DM. Carbon Ion Therapy: A Modern Review of an Emerging Technology. Front Oncol (2020) 10:82. doi: 10.3389/fonc.2020.00082 32117737 PMC7010911

[10] Rackwitz T Debus J. Clinical Applications of Proton and Carbon Ion Therapy. Semin Oncol (2019) 46(3):226–32. doi: 10.1053/j.seminoncol.2019.07.005 31451309

[11] Jäkel O Schulz-Ertner D Karger CP Nikoghosyan A Debus J. Heavy Ion Therapy: Status and Perspectives. Technol Cancer Res Treat (2003) 2(5):377–87. doi: 10.1177/153303460300200503 14529303

[12] Lorat Y Reindl J Isermann A Rübe C Friedl AA Rübe CE. Focused Ion Microbeam Irradiation Induces Clustering of DNA Double-Strand Breaks in Heterochromatin Visualized by Nanoscale-Resolution Electron Microscopy. Int J Mol Sci (2021) 22(14):7638. doi: 10.3390/ijms22147638 34299263 PMC8306362

[13] Fossati P Matsufuji N Kamada T Karger CP. Radiobiological Issues in Prospective Carbon Ion Therapy Trials. Med Phys (2018) 45(11):e1096–110. doi: 10.1002/mp.12506 30421806

[14] Schlaff CD Krauze A Belard A O’Connell JJ Camphausen KA. Bringing the Heavy: Carbon Ion Therapy in the Radiobiological and Clinical Context. Radiat Oncol (2014) 9(1):88. doi: 10.1186/1748-717X-9-88 24679134 PMC4002206

[15] Ando K Kase Y. Biological Characteristics of Carbon-Ion Therapy. Int J Radiat Biol (2009) 85(9):715–28. doi: 10.1080/09553000903072470 19728191

[16] Atkinson JC Grisius M Massey W. Salivary Hypofunction and Xerostomia: Diagnosis and Treatment. Dent Clin North Am (2005) 49(2):309–26. doi: 10.1016/j.cden.2004.10.002 15755407

[17] Khaw A Logan R Keefe D Bartold M. Radiation-Induced Oral Mucositis and Periodontitis - Proposal for an Inter-Relationship. Oral Dis (2014) 20(3):e7–18. doi: 10.1111/odi.12199 24147592

[18] Peters M Hoekstra CJ van Voort Zyp JRN Westendorp H van de Pol SMG Moerland MA. Rectal Dose Constraints for Salvage Iodine-125 Prostate Brachytherapy. Brachytherapy (2016) 15(1):85–93. doi: 10.1016/j.brachy.2015.10.004 26614233

[19] You SH Cho MY Sohn JH Lee CG. Pancreatic Radiation Effect in Apoptosis-Related Rectal Radiation Toxicity. J Radiat Res (2018) 59(5):529–40. doi: 10.1093/jrr/rry043 PMC6151648 29901726

[20] Pan YB Maeda Y Wilson A Glynne-Jones R Vaizey CJ. Late Gastrointestinal Toxicity After Radiotherapy for Anal Cancer: A Systematic Literature Review. Acta Oncol (2018) 57(11):1427–37. doi: 10.1080/0284186X.2018.1503713 30264638

[21] Alterio D Gerardi MA Cella L Spoto R Zurlo V Sabbatini A. Strahleninduzierte Akute Dysphagie: Prospektive Beobachtungsstudie an 42 Kopf-Hals-Malignompatienten. Strahlenther Onkol (2017) 193(11):971–81. doi: 10.1007/s00066-017-1206-x 28884310

[22] Abdulla O White E. Radiation-Induced Sigmoid Stricture: An Important Differential. Br J Hosp Med (Lond) (2017) 78(11):654. doi: 10.12968/hmed.2017.78.11.654a 29111792

[23] Bresolin A Faiella A Garibaldi E Munoz F Cante D Vavassori V. Acute Patient-Reported Intestinal Toxicity in Whole Pelvis IMRT for Prostate Cancer: Bowel Dose-Volume Effect Quantification in a Multicentric Cohort Study. Radiother Oncol (2021) 158:74–82. doi: 10.1016/j.radonc.2021.02.026 33639190

[24] Ozkaya Akagunduz O Eyigor S Kirakli E Tavlayan E Erdogan Cetin Z Kara G. Radiation-Associated Chronic Dysphagia Assessment by Flexible Endoscopic Evaluation of Swallowing (FEES) in Head and Neck Cancer Patients: Swallowing-Related Structures and Radiation Dose-Volume Effect. Ann Otol Rhinol Laryngol (2019) 128(2):73–84. doi: 10.1177/0003489418804260 30343589

[25] Carrington R Staffurth J Warren S Partridge M Hurt C Spezi E. The Effect of Dose Escalation on Gastric Toxicity When Treating Lower Oesophageal Tumours: A Radiobiological Investigation. Radiat Oncol (2015) 10:236. doi: 10.1186/s13014-015-0537-y 26586375 PMC4653919

[26] Kavanagh BD Pan CC Dawson LA Das SK Li XA ten Haken RK. Radiation Dose-Volume Effects in the Stomach and Small Bowel. Int J Radiat Oncol Biol Phys (2010) 76(3 Suppl):S101–7. doi: 10.1016/j.ijrobp.2009.05.071 20171503

[27] Li Q Chen J Zhu B Jiang M Liu W Lu E. Dose Volume Effect of Acute Diarrhea in Post-Operative Radiation for Gynecologic Cancer. Rev Invest Clin (2017) 69(6):329–35. doi: 10.24875/RIC.17002373 29265117

[28] Thor M Olsson CE Oh JH Petersen SE Alsadius D Bentzen L. Relationships Between Dose to the Gastro-Intestinal Tract and Patient-Reported Symptom Domains After Radiotherapy for Localized Prostate Cancer. Acta Oncol (2015) 54(9):1326–34. doi: 10.3109/0284186X.2015.1063779 PMC4786008 26340136

[29] Thor M Jackson A Zelefsky MJ Steineck G Karlsdòttir A Høyer M. Inter-Institutional Analysis Demonstrates the Importance of Lower Than Previously Anticipated Dose Regions to Prevent Late Rectal Bleeding Following Prostate Radiotherapy. Radiother Oncol (2018) 127(1):88–95. doi: 10.1016/j.radonc.2018.02.020 29530433 PMC6628908

[30] Chicas-Sett R Farga D Perez-Calatayud MJ Celada F Roldan S Fornes-Ferrer V. High-Dose-Rate Brachytherapy Boost for Prostate Cancer: Analysis of Dose-Volume Histogram Parameters for Predicting Late Rectal Toxicity. Brachytherapy (2017) 16(3):511–7. doi: 10.1016/j.brachy.2017.03.002 28366276

[31] Taniguchi T Iinuma K Kato D Takai M Maekawa YM Nakane K. Predictive Factors of Rectal Hemorrhage in Patients With Localized Prostate Cancer Who Underwent Low-Dose-Rate Brachytherapy. Int J Clin Oncol (2020) 25(9):1711–7. doi: 10.1007/s10147-020-01713-x 32500469

[32] Holyoake DLP Warren DR Hurt C Aznar M Partridge M Mukherjee S. Stomach Dose-Volume Predicts Acute Gastrointestinal Toxicity in Chemoradiotherapy for Locally Advanced Pancreatic Cancer. Clin Oncol (R Coll Radiol) (2018) 30(7):418–26. doi: 10.1016/j.clon.2018.02.067 29602584

[33] Casares-Magaz O Muren LP Moiseenko V Petersen SE Pettersson NJ Høyer M. Spatial Rectal Dose/Volume Metrics Predict Patient-Reported Gastro-Intestinal Symptoms After Radiotherapy for Prostate Cancer. Acta Oncol (2017) 56(11):1507–13. doi: 10.1080/0284186X.2017.1370130 PMC6619498 28885095

[34] Peng X Zhou S Liu S Li J Huang S Jiang X. Dose-Volume Analysis of Predictors for Acute Anal Toxicity After Radiotherapy in Prostate Cancer Patients. Radiat Oncol (2019) 14(1):174. doi: 10.1186/s13014-019-1374-1 31601249 PMC6785897

[35] Kim JW Kim JM Choi ME Kim S-K Kim Y-M Choi J-S. Does Salivary Function Decrease in Proportion to Radioiodine Dose? Laryngoscope (2020) 130(9):2173–8. doi: 10.1002/lary.28342 31765488

[36] Scaife JE Thomas SJ Harrison K Romanchikova M Sutcliffe MPF Forman JR. Accumulated Dose to the Rectum, Measured Using Dose-Volume Histograms and Dose-Surface Maps, Is Different From Planned Dose in All Patients Treated With Radiotherapy for Prostate Cancer. Br J Radiol (2015) 88(1054):20150243. doi: 10.1259/bjr.20150243 26204919 PMC4730972

[37] Wang K Pearlstein KA Moon DH Mahbooba ZM Deal AM Wang Y. Assessment of Risk of Xerostomia After Whole-Brain Radiation Therapy and Association With Parotid Dose. JAMA Oncol (2019) 5(2):221–8. doi: 10.1001/jamaoncol.2018.4951 PMC6439567 30489607

[38] Romano E Simon R Minard-Colin V Martin V Bockel S Espenel S. Analysis of Radiation Dose/Volume Effect Relationship for Anorectal Morbidity in Children Treated for Pelvic Malignancies. Int J Radiat Oncol Biol Phys (2021) 109(1):231–41. doi: 10.1016/j.ijrobp.2020.08.033 32805302

[39] Li X Xiao C Kong Y Guo W Zhan W Li G. Rectal Wall Dose-Volume Effect of Pre- or Post KUSHEN Ningjiaos Relationship With 3D Brachytherapy in Cervical Cancer Patients. Radiat Oncol (2019) 14(1):149. doi: 10.1186/s13014-019-1354-5 31429773 PMC6700783

[40] Mazeron R Fokdal LU Kirchheiner K Georg P Jastaniyah N Šegedin B. Dose-Volume Effect Relationships for Late Rectal Morbidity in Patients Treated With Chemoradiation and MRI-Guided Adaptive Brachytherapy for Locally Advanced Cervical Cancer: Results From the Prospective Multicenter EMBRACE Study. Radiother Oncol (2016) 120(3):412–9. doi: 10.1016/j.radonc.2016.06.006 27396811

[41] Mazeron R Maroun P Castelnau-Marchand P Dumas I Del Campo ER Cao K. Pulsed-Dose Rate Image-Guided Adaptive Brachytherapy in Cervical Cancer: Dose-Volume Effect Relationships for the Rectum and Bladder. Radiother Oncol (2015) 116(2):226–32. doi: 10.1016/j.radonc.2015.06.027 26164773

[42] Sun X Chen A Xie C Jin X Wu S-X Zhang P. The Relationship Between the Parotid Glands Function and the Dose-Volume Effect in Nasopharyngeal Carcinoma Patients With Intensity-Modulated Radiation Therapy. Zhonghua Yi Xue Za Zhi (2006) 86(32):2289–92. doi: 10.3760/j:issn:0376-2491.2006.32.014 17064579

[43] Zapatero A García-Vicente F Modolell I Alcántara P Floriano A Cruz-Conde A. Impact of Mean Rectal Dose on Late Rectal Bleeding After Conformal Radiotherapy for Prostate Cancer: Dose-Volume Effect. Int J Radiat Oncol Biol Phys (2004) 59(5):1343–51. doi: 10.1016/j.ijrobp.2004.01.031 15275719

[44] Huang J Robertson JM Ye H Margolis J Nadeau L Yan D. Dose-Volume Analysis of Predictors for Gastrointestinal Toxicity After Concurrent Full-Dose Gemcitabine and Radiotherapy for Locally Advanced Pancreatic Adenocarcinoma. Int J Radiat Oncol Biol Phys (2012) 83(4):1120–5. doi: 10.1016/j.ijrobp.2011.09.022 22099048

[45] Kim JW Kim TH Kim J-H Lee IJ. Predictors of Post-Treatment Stenosis in Cervical Esophageal Cancer Undergoing High-Dose Radiotherapy. World J Gastroenterol (2018) 24(7):862–9. doi: 10.3748/wjg.v24.i7.862 PMC5807944 29467556

[46] Jiang L Huang C Gan Y Wu T Tang X Wang Y. Radiation-Induced Late Dysphagia After Intensity-Modulated Radiotherapy in Nasopharyngeal Carcinoma Patients: A Dose-Volume Effect Analysis. Sci Rep (2018) 8(1):16396. doi: 10.1038/s41598-018-34803-y 30401941 PMC6219576

[47] Scalco E Fiorino C Cattaneo GM Sanguineti G Rizzo G. Texture Analysis for the Assessment of Structural Changes in Parotid Glands Induced by Radiotherapy. Radiother Oncol (2013) 109(3):384–7. doi: 10.1016/j.radonc.2013.09.019 24183861

[48] Nabaa B Takahashi K Sasaki T Okizaki A Aburano T. Assessment of Salivary Gland Dysfunction After Radioiodine Therapy for Thyroid Carcinoma Using Non-Contrast-Enhanced CT: The Significance of Changes in Volume and Attenuation of the Glands. AJNR Am J Neuroradiol (2012) 33(10):1964–70. doi: 10.3174/ajnr.A3063 PMC7964601 22555571

[49] Belli ML Scalco E Sanguineti G Fiorino C Broggi S Dinapoli N. Early Changes of Parotid Density and Volume Predict Modifications at the End of Therapy and Intensity of Acute Xerostomia. Strahlenther Onkol (2014) 190(11):1001–7. doi: 10.1007/s00066-014-0669-2 24756139

[50] Wu H Chen X Yang X Tao Y Xia Y Deng X. Early Prediction of Acute Xerostomia During Radiation Therapy for Head and Neck Cancer Based on Texture Analysis of Daily CT. Int J Radiat Oncol Biol Phys (2018) 102(4):1308–18. doi: 10.1016/j.ijrobp.2018.04.059 29891201

[51] van Dijk LV Noordzij W Brouwer CL Boellaard R Burgerhof JGM Langendijk JA. 18f-FDG PET Image Biomarkers Improve Prediction of Late Radiation-Induced Xerostomia. Radiother Oncol (2018) 126(1):89–95. doi: 10.1016/j.radonc.2017.08.024 28951007

[52] Rabe TM Yokoo T Meyer J Kernstine KH Wang D Khatri G. Radiation-Induced Liver Injury Mimicking Metastatic Disease in a Patient With Esophageal Cancer: Correlation of Positron Emission Tomography/Computed Tomography With Magnetic Resonance Imaging and Literature Review. J Comput Assist Tomogr (2016) 40(4):560–3. doi: 10.1097/RCT.0000000000000406 27023857

[53] Solomon J Marin D Roy Choudhury K Patel B Samei E. Effect of Radiation Dose Reduction and Reconstruction Algorithm on Image Noise, Contrast, Resolution, and Detectability of Subtle Hypoattenuating Liver Lesions at Multidetector CT: Filtered Back Projection Versus a Commercial Model-Based Iterative Reconstruction Algorithm. Radiology (2017) 284(3):777–87. doi: 10.1148/radiol.2017161736 PMC5702911 28170300

[54] Kabarriti R Brodin NP Yaffe H Barahman M Koba WR Liu L. Non-Invasive Targeted Hepatic Irradiation and SPECT/CT Functional Imaging to Study Radiation-Induced Liver Damage in Small Animal Models. Cancers (Basel) (2019) 11(11):1796. doi: 10.3390/cancers11111796 PMC6896151 31731687

[55] Yang X Tridandapani S Beitler JJ Yu DS Yoshida EJ Curran WJ. Ultrasound GLCM Texture Analysis of Radiation-Induced Parotid-Gland Injury in Head-and-Neck Cancer Radiotherapy: An In Vivo Study of Late Toxicity. Med Phys (2012) 39(9):5732–9. doi: 10.1118/1.4747526 PMC3443195 22957638

[56] Yang X Tridandapani S Beitler JJ Yu DS Yoshida EJ Curran WJ. Ultrasound Histogram Assessment of Parotid Gland Injury Following Head-and-Neck Radiotherapy: A Feasibility Study. Ultrasound Med Biol (2012) 38(9):1514–21. doi: 10.1016/j.ultrasmedbio.2012.05.005 PMC3633493 22766120

[57] Yang X Tridandapani S Beitler JJ Yu DS Chen Z Kim S. Diagnostic Accuracy of Ultrasonic Histogram Features to Evaluate Radiation Toxicity of the Parotid Glands: A Clinical Study of Xerostomia Following Head-and-Neck Cancer Radiotherapy. Acad Radiol (2014) 21(10):1304–13. doi: 10.1016/j.acra.2014.05.017 PMC4345172 25088832

[58] Casares-Magaz O Thor M Liao D Frøkjær JB Kræmer P Krogh K. An Image-Based Method to Quantify Biomechanical Properties of the Rectum in Radiotherapy of Prostate Cancer. Acta Oncol (2015) 54(9):1335–42. doi: 10.3109/0284186X.2015.1066933 PMC6628909 26198656

[59] Jelvehgaran P Steinberg JD Khmelinskii A Borst G Song J-Y de Wit N. Evaluation of Acute Esophageal Radiation-Induced Damage Using Magnetic Resonance Imaging: A Feasibility Study in Mice. Radiat Oncol (2019) 14(1):188. doi: 10.1186/s13014-019-1396-8 31666092 PMC6822441

[60] Marzi S Farneti A Vidiri A Di Giuliano F Marucci L Spasiano F. Radiation-Induced Parotid Changes in Oropharyngeal Cancer Patients: The Role of Early Functional Imaging and Patient-/Treatment-Related Factors. Radiat Oncol (2018) 13(1):189. doi: 10.1186/s13014-018-1137-4 30285893 PMC6167883

[61] van Dijk LV Thor M Steenbakkers RJHM Apte A Zhai T-T Borra R. Parotid Gland Fat Related Magnetic Resonance Image Biomarkers Improve Prediction of Late Radiation-Induced Xerostomia. Radiother Oncol (2018) 128(3):459–66. doi: 10.1016/j.radonc.2018.06.012 PMC6625348 29958772

[62] Chen D-C Chen L-H Jin W-D Xu Y-K Xu P-J. Magnetic Resonance Imaging Findings of Liver Injury Induced by Three-Dimensional Conformal Radiotherapy. Nan Fang Yi Ke Da Xue Xue Bao (2007) 27(2):181–3, 187. 17355931

[63] Lee J Han HJ Min BS Hong SP Shin SJ Yoon H. The Role of Endoscopic Evaluation for Radiation Proctitis in Patients Receiving Intermediate-Dose Postoperative Radiotherapy for Rectal Cancer. Jpn J Clin Oncol (2018) 48(11):988–94. doi: 10.1093/jjco/hyy126 30239826

[64] Ruiz-Rebollo ML de-la-Calle F Velayos B Fernández-Salazar L Aller-de-la-Fuente R González JM. Radiation Enteritidis Diagnosed by Wireless Capsule Endoscopy. Rev Esp Enferm Dig (2012) 104(4):212–3. doi: 10.4321/s1130-01082012000400008 22537371

[65] Kopelman Y Groissman G Fireman Z. Radiation Enteritis Diagnosed by Capsule Endoscopy. Gastrointest Endosc (2007) 66(3):599; discussion 599. doi: 10.1016/j.gie.2007.03.006 17725954

[66] Lee JK Agrawal D Thosani N Al-Haddad M Buxbaum JL Calderwood AH. ASGE Guideline on the Role of Endoscopy for Bleeding From Chronic Radiation Proctopathy. Gastrointest Endosc (2019) 90(2):171–182.e1. doi: 10.1016/j.gie.2019.04.234 31235260

[67] Won S-M Park E Jung J-J Ganesan R Gupta H Gebru YA. The Gut Microbiota-Derived Immune Response in Chronic Liver Disease. Int J Mol Sci (2021) 22(15):8309. doi: 10.3390/ijms22158309 34361075 PMC8347749

[68] Cunningham AL Stephens JW Harris DA. Gut Microbiota Influence in Type 2 Diabetes Mellitus (T2DM). Gut Pathog (2021) 13(1):50. doi: 10.1186/s13099-021-00446-0 34362432 PMC8343927

[69] Nardone OM de Sire R Petito V Testa A Villani G Scaldaferri F. Inflammatory Bowel Diseases and Sarcopenia: The Role of Inflammation and Gut Microbiota in the Development of Muscle Failure. Front Immunol (2021) 12:694217. doi: 10.3389/fimmu.2021.694217 34326845 PMC8313891

[70] Li Q Gao B Siqin B He Q Zhang R Meng X. Gut Microbiota: A Novel Regulator of Cardiovascular Disease and Key Factor in the Therapeutic Effects of Flavonoids. Front Pharmacol (2021) 12:651926. doi: 10.3389/fphar.2021.651926 34220497 PMC8241904

[71] de Marco Castro E Murphy CH Roche HM. Targeting the Gut Microbiota to Improve Dietary Protein Efficacy to Mitigate Sarcopenia. Front Nutr (2021) 8:656730. doi: 10.3389/fnut.2021.656730 34235167 PMC8256992

[72] Jaye K Li CG Bhuyan DJ. The Complex Interplay of Gut Microbiota With the Five Most Common Cancer Types: From Carcinogenesis to Therapeutics to Prognoses. Crit Rev Oncol Hematol (2021) 165:103429. doi: 10.1016/j.critrevonc.2021.103429 34293459

[73] Sims TT El Alam MB Karpinets TV Dorta-Estremera S Hegde VL Nookala S. Gut Microbiome Diversity is an Independent Predictor of Survival in Cervical Cancer Patients Receiving Chemoradiation. Commun Biol (2021) 4(1):237. doi: 10.1038/s42003-021-01741-x 33619320 PMC7900251

[74] Maier I Schiestl RH. Evidence From Animal Models: Is a Restricted or Conventional Intestinal Microbiota Composition Predisposing to Risk for High-LET Radiation Injury? Radiat Res (2015) 183(6):589–93. doi: 10.1667/RR13837.1 26010710

[75] Suzuki F Loucas BD Ito I Asai A Suzuki S Kobayashi M. Survival of Mice With Gastrointestinal Acute Radiation Syndrome Through Control of Bacterial Translocation. J Immunol (2018) 201(1):77–86. doi: 10.4049/jimmunol.1701515 29743312 PMC6008223

[76] Broin P Ó Vaitheesvaran B Saha S Hartil K Chen EI Goldman D. Intestinal Microbiota-Derived Metabolomic Blood Plasma Markers for Prior Radiation Injury. Int J Radiat Oncol Biol Phys (2015) 91(2):360–7. doi: 10.1016/j.ijrobp.2014.10.023 PMC4312583 25636760

[77] Chai Y Wang J Wang T Yang Y Su J Shi F. Application of 1H NMR Spectroscopy-Based Metabonomics to Feces of Cervical Cancer Patients With Radiation-Induced Acute Intestinal Symptoms. Radiother Oncol (2015) 117(2):294–301. doi: 10.1016/j.radonc.2015.07.037 26277430

[78] Campostrini F Remo A Astati L Zorzi M Capodaglio G Buffoli A. Assoziation Zwischen Akuten Histopathologischen Veränderungen Der Rektumwände Und Einer Späten Radiogenen Proktitis Nach Strahlentherapie Des Prostatakarzinoms. Strahlenther Onkol (2020) 196(7):617–27. doi: 10.1016/j.ijrobp.2017.01.008 32166451

[79] Coates J Jeyaseelan AK Ybarra N David M Faria S Souhami L. Contrasting Analytical and Data-Driven Frameworks for Radiogenomic Modeling of Normal Tissue Toxicities in Prostate Cancer. Radiother Oncol (2015) 115(1):107–13. doi: 10.1016/j.radonc.2015.03.005 25818395

[80] Ghorbanzadeh-Moghaddam A Gholamrezaei A Hemati S. Vitamin D Deficiency Is Associated With the Severity of Radiation-Induced Proctitis in Cancer Patients. Int J Radiat Oncol Biol Phys (2015) 92(3):613–8. doi: 10.1016/j.ijrobp.2015.02.011 25890844

[81] Onal C Kotek A Unal B Arslan G Yavuz A Topkan E. Plasma Citrulline Levels Predict Intestinal Toxicity in Patients Treated With Pelvic Radiotherapy. Acta Oncol (2011) 50(8):1167–74. doi: 10.3109/0284186X.2011.584557 21864050

[82] Arrifin A Heidari E Burke M Fenlon MR Banerjee A. The Effect of Radiotherapy for Treatment of Head and Neck Cancer on Oral Flora and Saliva. Oral Health Prev Dent (2018) 16(5):425–9. doi: 10.3290/j.ohpd.a41364 30460355

[83] Epstein JB Chin EA Jacobson JJ Rishiraj B Le N. The Relationships Among Fluoride, Cariogenic Oral Flora, and Salivary Flow Rate During Radiation Therapy. Oral Surg Oral Med Oral Pathol Oral Radiol Endod (1998) 86(3):286–92. doi: 10.1016/s1079-2104(98)90173-1 9768416

[84] Wu F Weng S Li C Sun J Li L Gao Q. Submandibular Gland Transfer for the Prevention of Postradiation Xerostomia in Patients With Head and Neck Cancer: A Systematic Review and Meta-Analysis. ORL J Otorhinolaryngol Relat Spec (2015) 77(2):70–86. doi: 10.1159/000371854 25823449

[85] Zhang X Liu F Lan X Yu L Wu W Wu X. Clinical Observation of Submandibular Gland Transfer for the Prevention of Xerostomia After Radiotherapy for Nasopharyngeal Carcinoma: A Prospective Randomized Controlled Study of 32 Cases. Radiat Oncol (2014) 9:62. doi: 10.1186/1748-717X-9-62 24555575 PMC3984745

[86] Zhang Y Guo C-B Zhang L Wang Y Peng X Mao C. Prevention of Radiation-Induced Xerostomia by Submandibular Gland Transfer. Head Neck (2012) 34(7):937–42. doi: 10.1002/hed.21859 22083885

[87] Jha N Harris J Seikaly H Jacobs JR McEwan AJB Robbins KT. A Phase II Study of Submandibular Gland Transfer Prior to Radiation for Prevention of Radiation-Induced Xerostomia in Head-and-Neck Cancer (RTOG 0244). Int J Radiat Oncol Biol Phys (2012) 84(2):437–42. doi: 10.1016/j.ijrobp.2012.02.034 PMC5746194 22541957

[88] Jha N Seikaly H Harris J Williams D Sultanem K Hier M. Phase III Randomized Study: Oral Pilocarpine Versus Submandibular Salivary Gland Transfer Protocol for the Management of Radiation-Induced Xerostomia. Head Neck (2009) 31(2):234–43. doi: 10.1002/hed.20961 19107948

[89] Sood AJ Fox NF O’Connell BP Lovelace TL Nguyen SA Sharma AK. Salivary Gland Transfer to Prevent Radiation-Induced Xerostomia: A Systematic Review and Meta-Analysis. Oral Oncol (2014) 50(2):77–83. doi: 10.1016/j.oraloncology.2013.10.010 24189058

[90] Accardi MV Donini O Rumage A Ascah A Haruna J Pouliot M. Characterization of a Partial-Body Irradiation Model With Oral Cavity Shielding in Nonhuman Primates. Int J Radiat Biol (2020) 96(1):100–11. doi: 10.1080/09553002.2018.1440093 29447591

[91] Rao AD Coquia S Jong R Gourin C Page B Latronico D. Effects of Biodegradable Hydrogel Spacer Injection on Contralateral Submandibular Gland Sparing in Radiotherapy for Head and Neck Cancers. Radiother Oncol (2018) 126(1):96–9. doi: 10.1016/j.radonc.2017.09.017 28985953

[92] Rucinski A Brons S Richter D Habl G Debus J Bert C. Ion Therapy of Prostate Cancer: Daily Rectal Dose Reduction by Application of Spacer Gel. Radiat Oncol (2015) 10:56. doi: 10.1186/s13014-015-0348-1 25886457 PMC4399750

[93] van Wijk Y Vanneste BGL Walsh S van der Meer S Ramaekers B van Elmpt W. Development of a Virtual Spacer to Support the Decision for the Placement of an Implantable Rectum Spacer for Prostate Cancer Radiotherapy: Comparison of Dose, Toxicity and Cost-Effectiveness. Radiother Oncol (2017) 125(1):107–12. doi: 10.1016/j.radonc.2017.07.026 28823404

[94] Rao AD Feng Z Shin EJ He J Waters KM Coquia S. A Novel Absorbable Radiopaque Hydrogel Spacer to Separate the Head of the Pancreas and Duodenum in Radiation Therapy for Pancreatic Cancer. Int J Radiat Oncol Biol Phys (2017) 99(5):1111–20. doi: 10.1016/j.ijrobp.2017.08.006 PMC5699940 28943075

[95] Pinkawa M Berneking V Schlenter M Krenkel B Eble MJ. Quality of Life After Radiation Therapy for Prostate Cancer With a Hydrogel Spacer: 5-Year Results. Int J Radiat Oncol Biol Phys (2017) 99(2):374–7. doi: 10.1016/j.ijrobp.2017.05.035 28871986

[96] Chao M Ho H Chan Y Tan A Pham T Bolton D. Prospective Analysis of Hydrogel Spacer for Patients With Prostate Cancer Undergoing Radiotherapy. BJU Int (2018) 122(3):427–33. doi: 10.1111/bju.14192 29520983

[97] Cirillo G Spizzirri UG Curcio M Nicoletta FP Iemma F. Injectable Hydrogels for Cancer Therapy Over the Last Decade. Pharmaceutics (2019) 11(9):486. doi: 10.3390/pharmaceutics11090486 PMC6781516 31546921

[98] Pang L Tian P Cui X Wu X Zhao X Wang H. *In Situ* Photo-Cross-Linking Hydrogel Accelerates Diabetic Wound Healing Through Restored Hypoxia-Inducible Factor 1-Alpha Pathway and Regulated Inflammation. ACS Appl Mater Interfaces (2021) 13(25):29363–79. doi: 10.1021/acsami.1c07103 34128630

[99] Pandey M Choudhury H D/O Segar Singh SK Chetty Annan N Bhattamisra SK Gorain B. Budesonide-Loaded Pectin/Polyacrylamide Hydrogel for Sustained Delivery: Fabrication, Characterization and *In Vitro* Release Kinetics. Molecules (2021) 26(9):2704. doi: 10.3390/molecules26092704 34062995 PMC8124457

[100] Qiao Y Zhang Q Wang Q Li Y Wang L. Filament-Anchored Hydrogel Layer on Polypropylene Hernia Mesh With Robust Anti-Inflammatory Effects. Acta Biomater (2021) 128:277–90. doi: 10.1016/j.actbio.2021.04.013 33866036

[101] Machado VS Camponogara C Oliveira SM Baldissera MD Sagrillo MR Da Gundel SS. Topical Hydrogel Containing Achyrocline Satureioides Oily Extract (Free and Nanocapsule) has Anti-Inflammatory Effects and Thereby Minimizes Irritant Contact Dermatitis. Acad Bras Cienc (2020) 92(4):e20191066. doi: 10.1590/0001-3765202020191066 33206785

[102] Wang Q-S Xu B-X Fan K-J Li Y-W Wu J Wang T-Y. Dexamethasone-Loaded Thermosensitive Hydrogel Suppresses Inflammation and Pain in Collagen-Induced Arthritis Rats. Drug Des Devel Ther (2020) 14:4101–13. doi: 10.2147/DDDT.S256850 PMC7547127 33116399

[103] Hauswald H Kamrava MR Fallon JM Wang P-C Park S-J Van T. High-Dose-Rate Monotherapy for Localized Prostate Cancer: 10-Year Results. Int J Radiat Oncol Biol Phys (2016) 94(4):667–74. doi: 10.1016/j.ijrobp.2015.07.2290 26443877

[104] Kragelj B Zlatic J Zaletel-Kragelj L. Avoidance of Late Rectal Toxicity After High-Dose-Rate Brachytherapy Boost Treatment for Prostate Cancer. Brachytherapy (2017) 16(1):193–200. doi: 10.1016/j.brachy.2016.10.008 27908678

[105] Fransson P Nilsson P Gunnlaugsson A Beckman L Tavelin B Norman D. Ultra-Hypofractionated Versus Conventionally Fractionated Radiotherapy for Prostate Cancer (HYPO-RT-PC): Patient-Reported Quality-of-Life Outcomes of a Randomised, Controlled, Non-inferiority, Phase 3 Trial. Lancet Oncol (2021) 22(2):235–45. doi: 10.1016/S1470-2045(20)30581-7 33444529

[106] Di Franco R Borzillo V Ravo V Ametrano G Cammarota F Rossetti S. Rectal/urinary Toxicity After Hypofractionated vs. Conventional Radiotherapy in High Risk Prostate Cancer: Systematic Review and Meta Analysis. Eur Rev Med Pharmacol Sci (2017) 21(16):3563–75. doi: 10.26355/eurrev_201708_13266 28925488

[107] Yin Z You J Wang Y Zhao J Jiang S Zhang X. Moderate Hypofractionated Radiotherapy vs Conventional Fractionated Radiotherapy in Localized Prostate Cancer: A Systemic Review and Meta-Analysis From Phase III Randomized Trials. Onco Targets Ther (2019) 12:1259–68. doi: 10.2147/OTT.S181067 PMC6388980 30863093

[108] Yoon SM Chu F-I Ruan D Steinberg ML Raldow A Lee P. Assessment of Toxic Effects Associated With Dose-Fractionated Radiotherapy Among Patients With Cancer and Comorbid Collagen Vascular Disease. JAMA Netw Open (2021) 4(2):e2034074. doi: 10.1001/jamanetworkopen.2020.34074 33599771 PMC7893499

[109] Kim D-Y Park E Heo CY Jin US Kim EK Han W. Hypofractionated Versus Conventional Fractionated Radiotherapy for Breast Cancer in Patients With Reconstructed Breast: Toxicity Analysis. Breast (2021) 55:37–44. doi: 10.1016/j.breast.2020.11.020 33316582 PMC7744765

[110] Wang S-L Fang H Hu C Song Y-W Wang W-H Jin J. Hypofractionated Versus Conventional Fractionated Radiotherapy After Breast-Conserving Surgery in the Modern Treatment Era: A Multicenter, Randomized Controlled Trial From China. J Clin Oncol (2020) 38(31):3604–14. doi: 10.1200/JCO.20.01024 32780661

[111] Liu L Yang Y Guo Q Ren B Peng Q Zou L. Comparing Hypofractionated to Conventional Fractionated Radiotherapy in Postmastectomy Breast Cancer: A Meta-Analysis and Systematic Review. Radiat Oncol (2020) 15(1):17. doi: 10.1186/s13014-020-1463-1 31952507 PMC6969477

[112] Widmark A Gunnlaugsson A Beckman L Thellenberg-Karlsson C Hoyer M Lagerlund M. Ultra-Hypofractionated Versus Conventionally Fractionated Radiotherapy for Prostate Cancer: 5-Year Outcomes of the HYPO-RT-PC Randomised, Non-Inferiority, Phase 3 Trial. Lancet (2019) 394(10196):385–95. doi: 10.1016/S0140-6736(19)31131-6 31227373

[113] Moussa L Demarquay C Réthoré G Benadjaoud MA Siñeriz F Pattapa G. Heparan Sulfate Mimetics: A New Way to Optimize Therapeutic Effects of Hydrogel-Embedded Mesenchymal Stromal Cells in Colonic Radiation-Induced Damage. Sci Rep (2019) 9(1):164. doi: 10.1038/s41598-018-36631-6 30655576 PMC6336771

[114] Niu S Zhang Y. Applications and Therapeutic Mechanisms of Action of Mesenchymal Stem Cells in Radiation-Induced Lung Injury. Stem Cell Res Ther (2021) 12(1):212. doi: 10.1186/s13287-021-02279-9 33766127 PMC7993004

[115] Kojima T Kanemaru S-I Hirano S Tateya I Ohno S Nakamura T. Regeneration of Radiation Damaged Salivary Glands With Adipose-Derived Stromal Cells. Laryngoscope (2011) 121(9):1864–9. doi: 10.1002/lary.22080 21748735

[116] Xiong X Shi X Chen F. Human Adipose Tissue−Derived Stem Cells Alleviate Radiation−Induced Xerostomia. Int J Mol Med (2014) 34(3):749–55. doi: 10.3892/ijmm.2014.1837 PMC4121343 25017690

[117] Jeong J Baek H Kim Y-J Choi Y Lee H Lee E. Human Salivary Gland Stem Cells Ameliorate Hyposalivation of Radiation-Damaged Rat Salivary Glands. Exp Mol Med (2013) 45:e58. doi: 10.1038/emm.2013.121 24232257 PMC3849572

[118] Nanduri LSY Lombaert IMA van der Zwaag M Faber H Brunsting JF van Os RP. Salisphere Derived C-Kit+ Cell Transplantation Restores Tissue Homeostasis in Irradiated Salivary Gland. Radiother Oncol (2013) 108(3):458–63. doi: 10.1016/j.radonc.2013.05.020 23769181

[119] Li Z Wang Y Xing H Wang Z Hu H An R. Protective Efficacy of Intravenous Transplantation of Adipose-Derived Stem Cells for the Prevention of Radiation-Induced Salivary Gland Damage. Arch Oral Biol (2015) 60(10):1488–96. doi: 10.1016/j.archoralbio.2015.07.016 26263537

[120] Grønhøj C Jensen DH Vester-Glowinski P Jensen SB Bardow A Oliveri RS. Safety and Efficacy of Mesenchymal Stem Cells for Radiation-Induced Xerostomia: A Randomized, Placebo-Controlled Phase 1/2 Trial (MESRIX). Int J Radiat Oncol Biol Phys (2018) 101(3):581–92. doi: 10.1016/j.ijrobp.2018.02.034 29678523

[121] Shin H-S Lee S Kim Y-M Lim J-Y. Hypoxia-Activated Adipose Mesenchymal Stem Cells Prevents Irradiation-Induced Salivary Hypofunction by Enhanced Paracrine Effect Through Fibroblast Growth Factor 10. Stem Cells (2018) 36(7):1020–32. doi: 10.1002/stem.2818 29569790

[122] Zhang C Zhang Y Feng Z Zhang F Liu Z Sun X. Therapeutic Effect of Dental Pulp Stem Cell Transplantation on a Rat Model of Radioactivity-Induced Esophageal Injury. Cell Death Dis (2018) 9(7):738. doi: 10.1038/s41419-018-0753-0 29970894 PMC6030227

[123] Durand C Pezet S Eutamène H Demarquay C Mathieu N Moussa L. Persistent Visceral Allodynia in Rats Exposed to Colorectal Irradiation Is Reversed by Mesenchymal Stromal Cell Treatment. Pain (2015) 156(8):1465–76. doi: 10.1097/j.pain.0000000000000190 25887464

[124] Linard C Busson E Holler V Strup-Perrot C Lacave-Lapalun J-V Lhomme B. Repeated Autologous Bone Marrow-Derived Mesenchymal Stem Cell Injections Improve Radiation-Induced Proctitis in Pigs. Stem Cells Transl Med (2013) 2(11):916–27. doi: 10.5966/sctm.2013-0030 PMC3808206 24068742

[125] Chen Y Niu Z Xue Y Yuan F Fu Y Bai N. Improvement in the Repair of Defects in Maxillofacial Soft Tissue in Irradiated Minipigs by a Mixture of Adipose-Derived Stem Cells and Platelet-Rich Fibrin. Br J Oral Maxillofac Surg (2014) 52(8):740–5. doi: 10.1016/j.bjoms.2014.06.006 24993354

[126] Nicolay NH Lopez Perez R Debus J Huber PE. Mesenchymal Stem Cells – A New Hope for Radiotherapy-Induced Tissue Damage? Cancer Lett (2015) 366(2):133–40. doi: 10.1016/j.canlet.2015.06.012 26166559

[127] Myung H Jang H Myung JK Lee C Lee J Kang J. Platelet-Rich Plasma Improves the Therapeutic Efficacy of Mesenchymal Stem Cells by Enhancing Their Secretion of Angiogenic Factors in a Combined Radiation and Wound Injury Model. Exp Dermatol (2020) 29(2):158–67. doi: 10.1111/exd.14042 31560791

[128] Chang P Qu Y Liu Y Cui S Zhu D Wang H. Multi-Therapeutic Effects of Human Adipose-Derived Mesenchymal Stem Cells on Radiation-Induced Intestinal Injury. Cell Death Dis (2013) 4:e685. doi: 10.1038/cddis.2013.178 23788042 PMC3698545

[129] Garg S Wang W Prabath BG Boerma M Wang J Zhou D. Bone Marrow Transplantation Helps Restore the Intestinal Mucosal Barrier After Total Body Irradiation in Mice. Radiat Res (2014) 181(3):229–39. doi: 10.1667/RR13548.1 PMC4038129 24568131

[130] Pejchal J Šinkorová Z Tichý A Kmochová A Ďurišová K Kubelková K. Attenuation of Radiation-Induced Gastrointestinal Damage by Epidermal Growth Factor and Bone Marrow Transplantation in Mice. Int J Radiat Biol (2015) 91(9):703–14. doi: 10.3109/09553002.2015.1054528 25994811

[131] I T Sumita Y Minamizato T Umebayashi M Liu Y Tran SD. Bone Marrow-Derived Cell Therapy for Oral Mucosal Repair After Irradiation. J Dent Res (2014) 93(8):813–20. doi: 10.1177/0022034514541124 PMC4293762 24980658

[132] Tran SD Liu Y Xia D Maria OM Khalili S Wang RW-J. Paracrine Effects of Bone Marrow Soup Restore Organ Function, Regeneration, and Repair in Salivary Glands Damaged by Irradiation. PloS One (2013) 8(4):e61632. doi: 10.1371/journal.pone.0061632 23637870 PMC3634855

[133] Chang YH Lin L-M Lou C-W Chou C-K Ch’ang H-J. Bone Marrow Transplantation Rescues Intestinal Mucosa After Whole Body Radiation *via* Paracrine Mechanisms. Radiother Oncol (2012) 105(3):371–7. doi: 10.1016/j.radonc.2012.10.005 23146318

[134] Touchefeu Y Montassier E Nieman K Gastinne T Potel G Des Bruley Varannes S. Systematic Review: The Role of the Gut Microbiota in Chemotherapy- or Radiation-Induced Gastrointestinal Mucositis - Current Evidence and Potential Clinical Applications. Aliment Pharmacol Ther (2014) 40(5):409–21. doi: 10.1111/apt.12878 25040088

[135] Guo H Chou WC Lai Y Liang K Tam JW Brickey WJ. Multi-Omics Analyses of Radiation Survivors Identify Radioprotective Microbes and Metabolites. Science (2020) 370(6516):eaay9097. doi: 10.1126/science.aay9097 33122357 PMC7898465

[136] Uribe-Herranz M Rafail S Beghi S Gil-de-Gómez L Verginadis I Bittinger K. Gut Microbiota Modulate Dendritic Cell Antigen Presentation and Radiotherapy-Induced Antitumor Immune Response. J Clin Invest (2020) 130(1):466–79. doi: 10.1172/JCI124332 PMC6934221 31815742

[137] Xiao HW Cui M Li Y Dong JL Zhang SQ Zhu CC. Gut Microbiota-Derived Indole 3-Propionic Acid Protects Against Radiation Toxicity *via* Retaining Acyl-CoA-Binding Protein. Microbiome (2020) 8(1):69. doi: 10.1186/s40168-020-00845-6 32434586 PMC7241002

[138] Zhang Y Dong Y Lu P Wang X Li W Dong H. Gut Metabolite Urolithin A Mitigates Ionizing Radiation-Induced Intestinal Damage. J Cell Mol Med (2021) p. 1–7. doi: 10.1111/jcmm.16951 PMC8572803 34595829

[139] Li Y Yan H Zhang Y Li Q Yu L Li Q. Alterations of the Gut Microbiome Composition and Lipid Metabolic Profile in Radiation Enteritis. Front Cell Infect Microbiol (2020) 10:541178. doi: 10.3389/fcimb.2020.541178 33194790 PMC7609817

[140] Then CK Paillas S Wang X Hampson A Kiltie AE. Association of Bacteroides Acidifaciens Relative Abundance With High-Fibre Diet-Associated Radiosensitisation. BMC Biol (2020) 18(1):102. doi: 10.1186/s12915-020-00836-x 32811478 PMC7437060

[141] Ferreira MR Muls A Dearnaley DP Andreyev HJN. Microbiota and Radiation-Induced Bowel Toxicity: Lessons From Inflammatory Bowel Disease for the Radiation Oncologist. Lancet Oncol (2014) 15(3):e139–47. doi: 10.1016/S1470-2045(13)70504-7 24599929

[142] Ki Y Kim W Cho H Ahn K Choi Y Kim D. The Effect of Probiotics for Preventing Radiation-Induced Morphological Changes in Intestinal Mucosa of Rats. J Korean Med Sci (2014) 29(10):1372–8. doi: 10.3346/jkms.2014.29.10.1372 PMC4214937 25368490

[143] Miousse IR Ewing LE Skinner CM Pathak R Garg S Kutanzi KR. Methionine Dietary Supplementation Potentiates Ionizing Radiation-Induced Gastrointestinal Syndrome. Am J Physiol Gastrointest Liver Physiol (2020) 318(3):G439–50. doi: 10.1152/ajpgi.00351.2019 PMC7099489 31961718

[144] Xiao H-W Li Y Luo D Dong J-L Zhou L-X Zhao S-Y. Hydrogen-Water Ameliorates Radiation-Induced Gastrointestinal Toxicity *via* Myd88’s Effects on the Gut Microbiota. Exp Mol Med (2018) 50(1):e433. doi: 10.1038/emm.2017.246 29371696 PMC5799803

[145] Zhang Y Zhang B Dong L Chang P. Potential of Omega-3 Polyunsaturated Fatty Acids in Managing Chemotherapy- or Radiotherapy-Related Intestinal Microbial Dysbiosis. Adv Nutr (2019) 10(1):133–47. doi: 10.1093/advances/nmy076 PMC6370266 30566596

[146] Cui M Xiao H Li Y Zhang S Dong J Wang B. Sexual Dimorphism of Gut Microbiota Dictates Therapeutics Efficacy of Radiation Injuries. Adv Sci (Weinh) (2019) 6(21):1901048. doi: 10.1002/advs.201901048 31728280 PMC6839645

[147] Reis Ferreira M Andreyev HJN Mohammed K Truelove L Gowan SM Li J. Microbiota- and Radiotherapy-Induced Gastrointestinal Side-Effects (MARS) Study: A Large Pilot Study of the Microbiome in Acute and Late-Radiation Enteropathy. Clin Cancer Res (2019) 25(21):6487–500. doi: 10.1158/1078-0432.CCR-19-0960 31345839

[148] Al-Qadami G van Sebille Y Le H Bowen J. Gut Microbiota: Implications for Radiotherapy Response and Radiotherapy-Induced Mucositis. Expert Rev Gastroenterol Hepatol (2019) 13(5):485–96. doi: 10.1080/17474124.2019.1595586 30907164

[149] Weiss JF Landauer MR. History and Development of Radiation-Protective Agents. Int J Radiat Biol (2009) 85(7):539–73. doi: 10.1080/09553000902985144 19557599

[150] Martin KL Hill GA Klein RR Arnett DG Burd R Limesand KH. Prevention of Radiation-Induced Salivary Gland Dysfunction Utilizing a CDK Inhibitor in a Mouse Model. PloS One (2012) 7(12):e51363. doi: 10.1371/journal.pone.0051363 23236487 PMC3517508

[151] Chang J Zhang H Guan F Wang Y Li D Wu H. The Protective Effects of SB203580 Against Mortality and Radiation Induced Intestinal Injury of Mice. Yao Xue Xue Bao (2011) 46(4):395–9. doi: 10.16438/j.0513-4870.2011.04.013 21751492

[152] Gheita HA El-Sabbagh WA Abdelsalam RM Attia AS El-Ghazaly MA. Promising Role of Filgrastim and α-Tocopherol Succinate in Amelioration of Gastrointestinal Acute Radiation Syndrome (GI-ARS) in Mice. Naunyn Schmiedebergs Arch Pharmacol (2019) 392(12):1537–50. doi: 10.1007/s00210-019-01702-6 31350581

[153] Matsuu-Matsuyama M Shichijo K Tsuchiya T Kondo H Miura S Matsuda K. Protective Effects of a Cystine and Theanine Mixture Against Acute Radiation Injury in Rats. Environ Toxicol Pharmacol (2020) 78:103395. doi: 10.1016/j.etap.2020.103395 32325407

[154] Bing SJ Kim MJ Ahn G Im J Kim DS Ha D. Acidic Polysaccharide of Panax Ginseng Regulates the Mitochondria/Caspase-Dependent Apoptotic Pathway in Radiation-Induced Damage to the Jejunum in Mice. Acta Histochem (2014) 116(3):514–21. doi: 10.1016/j.acthis.2013.11.012 24380494

[155] Chang JW Choi JW Lee BH Park JK Shin YS Oh Y-T. Protective Effects of Korean Red Ginseng on Radiation-Induced Oral Mucositis in a Preclinical Rat Model. Nutr Cancer (2014) 66(3):400–7. doi: 10.1080/01635581.2014.884234 24617451

[156] Gilman KE Camden JM Klein RR Zhang Q Weisman GA Limesand KH. P2X7 Receptor Deletion Suppresses γ-Radiation-Induced Hyposalivation. Am J Physiol Regul Integr Comp Physiol (2019) 316(5):R687–96. doi: 10.1152/ajpregu.00192.2018 PMC6589606 30892913

[157] He L-X Zhang Z-F Zhao J Li L Xu T Bin S. Ginseng Oligopeptides Protect Against Irradiation-Induced Immune Dysfunction and Intestinal Injury. Sci Rep (2018) 8(1):13916. doi: 10.1038/s41598-018-32188-6 30224720 PMC6141576

[158] Hou Q Liu L Dong Y Wu J Du L Dong H. Effects of Thymoquinone on Radiation Enteritis in Mice. Sci Rep (2018) 8(1):15122. doi: 10.1038/s41598-018-33214-3 30310156 PMC6181979

[159] Mercantepe F Topcu A Rakici S Tumkaya L Yilmaz A. The Effects of N-Acetylcysteine on Radiotherapy-Induced Small Intestinal Damage in Rats. Exp Biol Med (Maywood) (2019) 244(5):372–9. doi: 10.1177/1535370219831225 PMC6488866 30786762

[160] Purgason A Zhang Y Hamilton SR Gridley DS Sodipe A Jejelowo O. Apoptosis and Expression of Apoptosis-Related Genes in Mouse Intestinal Tissue After Whole-Body Proton Exposure. Mol Cell Biochem (2018) 442(1-2):155–68. doi: 10.1007/s11010-017-3200-0 29098506

[161] Son TG Gong EJ Bae MJ Kim SD Heo K Moon C. Protective Effect of Genistein on Radiation-Induced Intestinal Injury in Tumor Bearing Mice. BMC Complement Altern Med (2013) 13:103. doi: 10.1186/1472-6882-13-103 23672582 PMC3671128

[162] Pan W Hu L Chen Y Zhu Z Wang Y Song J. Sphingosine-1-Phosphate Alleviates Irradiation-Induced Parotid Injury in a Miniature Pig Model. Oral Dis (2020) 26(5):920–9. doi: 10.1111/odi.13302 32034858

[163] Staley EM Yarbrough VR Schoeb TR Daft JG Tanner SM Steverson D. Murine P-Glycoprotein Deficiency Alters Intestinal Injury Repair and Blunts Lipopolysaccharide-Induced Radioprotection. Radiat Res (2012) 178(3):207–16. doi: 10.1667/rr2835.1 PMC3474324 22780103

[164] Yang L Pan J. Therapeutic Effect of Ecdysterone Combine Paeonol Oral Cavity Direct Administered on Radiation-Induced Oral Mucositis in Rats. Int J Mol Sci (2019) 20(15):3800. doi: 10.3390/ijms20153800 PMC6695810 31382644

[165] Cagin YF Parlakpinar H Polat A Vardi N Atayan Y Erdogan MA. The Protective Effects of Apocynin on Ionizing Radiation-Induced Intestinal Damage in Rats. Drug Dev Ind Pharm (2016) 42(2):317–24. doi: 10.3109/03639045.2015.1052080 26072994

[166] Taniguchi CM Miao YR Diep AN Wu C Rankin EB Atwood TF. PHD Inhibition Mitigates and Protects Against Radiation-Induced Gastrointestinal Toxicity *via* HIF2. Sci Transl Med (2014) 6(236):236ra64. doi: 10.1126/scitranslmed.3008523 PMC4136475 24828078

[167] Zhang J Cui L Xu M Zheng Y. Restoring the Secretory Function of Irradiation-Damaged Salivary Gland by Administrating Deferoxamine in Mice. PloS One (2014) 9(11):e113721. doi: 10.1371/journal.pone.0113721 25427160 PMC4245233

[168] Lu L Jiang M Zhu C He J Fan S. Amelioration of Whole Abdominal Irradiation-Induced Intestinal Injury in Mice With 3,3’-Diindolylmethane (DIM). Free Radic Biol Med (2019) 130:244–55. doi: 10.1016/j.freeradbiomed.2018.10.410 30352304

[169] Yoon YJ Shin H-S Lim J-Y. A Hepatocyte Growth Factor/MET-Induced Antiapoptotic Pathway Protects Against Radiation-Induced Salivary Gland Dysfunction. Radiother Oncol (2019) 138:9–16. doi: 10.1016/j.radonc.2019.05.012 31136962

[170] Zhu N Liu R He L-X Mao R-X Liu X-R Zhang T. Radioprotective Effect of Walnut Oligopeptides Against Gamma Radiation-Induced Splenocyte Apoptosis and Intestinal Injury in Mice. Molecules (2019) 24(8):1582. doi: 10.3390/molecules24081582 PMC6515242 31013611

[171] Yang T Huang T Zhang D Wang M Wu B Shang Y. TGF-β Receptor Inhibitor LY2109761 Enhances the Radiosensitivity of Gastric Cancer by Inactivating the TGF-β/SMAD4 Signaling Pathway. Aging (Albany NY) (2019) 11(20):8892–910. doi: 10.18632/aging.102329 PMC6834415 31631064

[172] Lu C Cai D Ma J. Pachymic Acid Sensitizes Gastric Cancer Cells to Radiation Therapy by Upregulating Bax Through Hypoxia. Am J Chin Med (2018) 46(4):875–90. doi: 10.1142/S0192415X18500465 29737213

[173] Ghosh SP Kulkarni S Perkins MW Hieber K Pessu RL Gambles K. Amelioration of Radiation-Induced Hematopoietic and Gastrointestinal Damage by Ex-RAD(R) in Mice. J Radiat Res (2012) 53(4):526–36. doi: 10.1093/jrr/rrs001 PMC3393340 22843617

[174] Ha D Bing SJ Cho J Ahn G Kim DS Al-Amin M. Phloroglucinol Protects Small Intestines of Mice From Ionizing Radiation by Regulating Apoptosis-Related Molecules: A Comparative Immunohistochemical Study. J Histochem Cytochem (2013) 61(1):63–74. doi: 10.1369/0022155412468426 23117934 PMC3534317

[175] Khan S Kumar A Adhikari JS Rizvi MA Chaudhury NK. Protective Effect of Sesamol Against ⁶⁰Co γ-Ray-Induced Hematopoietic and Gastrointestinal Injury in C57BL/6 Male Mice. Free Radic Res (2015) 49(11):1344–61. doi: 10.3109/10715762.2015.1071485 26156438

[176] Li K Zhang J Cao J Li X Tian H. 1,4-Dithiothreitol Treatment Ameliorates Hematopoietic and Intestinal Injury in Irradiated Mice: Potential Application of a Treatment for Acute Radiation Syndrome. Int Immunopharmacol (2019) 76:105913. doi: 10.1016/j.intimp.2019.105913 31627170

[177] Li M Gu M-M Lang Y Shi J Chen BPC Guan H. The Vanillin Derivative VND3207 Protects Intestine Against Radiation Injury by Modulating P53/NOXA Signaling Pathway and Restoring the Balance of Gut Microbiota. Free Radic Biol Med (2019) 145:223–36. doi: 10.1016/j.freeradbiomed.2019.09.035 31580946

[178] Pant V Xiong S Wasylishen AR Larsson CA Aryal NK Chau G. Transient Enhancement of P53 Activity Protects From Radiation-Induced Gastrointestinal Toxicity. Proc Natl Acad Sci USA (2019) 116(35):17429–37. doi: 10.1073/pnas.1909550116 PMC6717251 31409715

[179] Venkateswaran K Shrivastava A Agrawala PK Prasad AK Devi SC Manda K. Mitigation of Radiation-Induced Gastro-Intestinal Injury by the Polyphenolic Acetate 7, 8-Diacetoxy-4-Methylthiocoumarin in Mice. Sci Rep (2019) 9(1):14134. doi: 10.1038/s41598-019-50785-x 31575959 PMC6773728

[180] Wang M Dong Y Wu J Li H Zhang Y Fan S. Baicalein Ameliorates Ionizing Radiation-Induced Injuries by Rebalancing Gut Microbiota and Inhibiting Apoptosis. Life Sci (2020) 261:118463. doi: 10.1016/j.lfs.2020.118463 32950576

[181] Huang E-Y Wang F-S Chen Y-M Chen Y-F Wang C-C Lin I-H. Amifostine Alleviates Radiation-Induced Lethal Small Bowel Damage via Promotion of 14-3-3ς-Mediated Nuclear P53 Accumulation. Oncotarget (2014) 5(20):9756–69. doi: 10.18632/oncotarget.2386 PMC4259435 25230151

[182] Mangoni M Sottili M Gerini C Fucci R Pini A Calosi L. Protective Effect of Leuprorelin on Radiation-Induced Intestinal Toxicity. Anticancer Res (2015) 35(7):3875–84. 26124333

[183] Wang X Wei L Cramer JM Leibowitz BJ Judge C Epperly M. Pharmacologically Blocking P53-Dependent Apoptosis Protects Intestinal Stem Cells and Mice From Radiation. Sci Rep (2015) 5:8566. doi: 10.1038/srep08566 25858503 PMC4392360

[184] Zhao DY Jacobs KM Hallahan DE Thotala D. Silencing Egr1 Attenuates Radiation-Induced Apoptosis in Normal Tissues While Killing Cancer Cells and Delaying Tumor Growth. Mol Cancer Ther (2015) 14(10):2343–52. doi: 10.1158/1535-7163.MCT-14-1051 PMC4845655 26206332

[185] Cheng Y Dong Y Hou Q Wu J Zhang W Tian H. The Protective Effects of XH-105 Against Radiation-Induced Intestinal Injury. J Cell Mol Med (2019) 23(3):2238–47. doi: 10.1111/jcmm.14159 PMC6378229 30663222

[186] Dong Y Cheng Y Hou Q Wu J Li D Tian H. The Protective Effect of New Compound XH-103 on Radiation-Induced GI Syndrome. Oxid Med Cell Longev (2018) 2018:3920147. doi: 10.1155/2018/3920147 30116481 PMC6079366

[187] Cho HT Kim JH Heo W Lee H-S Lee JJ Park T-S. Explosively Puffed Ginseng Ameliorates Ionizing Radiation-Induced Injury of Colon by Decreasing Oxidative Stress-Related Apoptotic Cell Execution in Mice. J Med Food (2019) 22(5):490–8. doi: 10.1089/jmf.2018.4293 31084541

[188] Nag D Bhanja P Riha R Sanchez-Guerrero G Kimler BF Tsue TT. Auranofin Protects Intestine Against Radiation Injury by Modulating P53/P21 Pathway and Radiosensitizes Human Colon Tumor. Clin Cancer Res (2019) 25(15):4791–807. doi: 10.1158/1078-0432.CCR-18-2751 PMC9159899 30940656

[189] Kandoth C McLellan MD Vandin F Ye K Niu B Lu C. Mutational Landscape and Significance Across 12 Major Cancer Types. Nature (2013) 502(7471):333–9. doi: 10.1038/nature12634 PMC3927368 24132290

[190] Helbling-Leclerc A Garcin C Rosselli F. Beyond DNA Repair and Chromosome Instability-Fanconi Anaemia as a Cellular Senescence-Associated Syndrome. Cell Death Differ (2021) 28(4):1159–73. doi: 10.1038/s41418-021-00764-5 PMC8026967 33723374

[191] Amigo JD Opazo JC Jorquera R Wichmann IA Garcia-Bloj BA Alarcon MA. The Reprimo Gene Family: A Novel Gene Lineage in Gastric Cancer With Tumor Suppressive Properties. Int J Mol Sci (2018) 19(7):1862. doi: 10.3390/ijms19071862 PMC6073456 29941787

[192] Bennett M Macdonald K Chan SW Luzio JP Simari R Weissberg P. Cell Surface Trafficking of Fas: A Rapid Mechanism of P53-Mediated Apoptosis. Science (1998) 282(5387):290–3. doi: 10.1126/science.282.5387.290 9765154

[193] Bock FJ Peintner L Tanzer M Manzl C Villunger A. P53-Induced Protein With a Death Domain (PIDD): Master of Puppets? Oncogene (2012) 31(45):4733–9. doi: 10.1038/onc.2011.639 PMC4562363 22266869

[194] Li L Han X Gao Y Diao Q Xiao Y. Ethanol Extract of Gynura Bicolor (GB) Protects Against UVB-Induced Photodamage of Skin by Inhibiting P53-Mediated Bcl-2/BAX/Caspase-3 Apoptosis Pathway. Arch Dermatol Res (2020) 312(1):41–9. doi: 10.1007/s00403-019-01977-y 31538224

[195] Mei Y Wu M. Multifaceted Functions of Siva-1: More Than an Indian God of Destruction. Protein Cell (2012) 3(2):117–22. doi: 10.1007/s13238-012-2018-5 PMC4875415 22426980

[196] Gupta RK Tripathi R Naidu BJ Srinivas UK Shashidhara LS. Cell Cycle Regulation by the Pro-Apoptotic Gene Scotin. Cell Cycle (2008) 7(15):2401–8. doi: 10.4161/cc.6407 18677103

[197] Tan J-H Cao R-C Zhou L Zhou Z-T Chen H-J Xu J. ATF6 Aggravates Acinar Cell Apoptosis and Injury by Regulating P53/AIFM2 Transcription in Severe Acute Pancreatitis. Theranostics (2020) 10(18):8298–314. doi: 10.7150/thno.46934 PMC7381726 32724472

[198] Leibowitz BJ Qiu W Liu H Cheng T Zhang L Yu J. Uncoupling P53 Functions in Radiation-Induced Intestinal Damage *via* PUMA and P21. Mol Cancer Res (2011) 9(5):616–25. doi: 10.1158/1541-7786.MCR-11-0052 PMC3096742 21450905

[199] Cao JY Dixon SJ. Mechanisms of Ferroptosis. Cell Mol Life Sci (2016) 73(11-12):2195–209. doi: 10.1007/s00018-016-2194-1 PMC4887533 27048822

[200] Stockwell BR Friedmann Angeli JP Bayir H Bush AI Conrad M Dixon SJ. Ferroptosis: A Regulated Cell Death Nexus Linking Metabolism, Redox Biology, and Disease. Cell (2017) 171(2):273–85. doi: 10.1016/j.cell.2017.09.021 PMC5685180 28985560

[201] Lang X Green MD Wang W Yu J Choi JE Jiang L. Radiotherapy and Immunotherapy Promote Tumoral Lipid Oxidation and Ferroptosis via Synergistic Repression of SLC7A11. Cancer Discov (2019) 9(12):1673–85. doi: 10.1158/2159-8290.CD-19-0338 PMC6891128 31554642

[202] Zhang X Xing X Liu H Feng J Tian M Chang S. Ionizing Radiation Induces Ferroptosis in Granulocyte-Macrophage Hematopoietic Progenitor Cells of Murine Bone Marrow. Int J Radiat Biol (2020) 96(5):584–95. doi: 10.1080/09553002.2020.1708993 31906761

[203] Yuan Z-H Liu T Wang H Xue L-X Wang J-J. Fatty Acids Metabolism: The Bridge Between Ferroptosis and Ionizing Radiation. Front Cell Dev Biol (2021) 9:675617. doi: 10.3389/fcell.2021.675617 34249928 PMC8264768

[204] Lei G Zhang Y Koppula P Liu X Zhang J Lin SH. The Role of Ferroptosis in Ionizing Radiation-Induced Cell Death and Tumor Suppression. Cell Res (2020) 30(2):146–62. doi: 10.1038/s41422-019-0263-3 PMC7015061 31949285

[205] Chen P-H Tseng WH-S Chi J-T. The Intersection of DNA Damage Response and Ferroptosis-A Rationale for Combination Therapeutics. Biol (Basel) (2020) 9(8):187. doi: 10.3390/biology9080187 PMC7464484 32718025

[206] Gan F Wang R Lyu P Li Y Fu R Du Y. Plasma-Derived Exosomes Boost the Healing of Irradiated Wound by Regulating Cell Proliferation and Ferroptosis. J BioMed Nanotechnol (2021) 17(1):100–14. doi: 10.1166/jbn.2021.3008 33653500

[207] Thermozier S Hou W Zhang X Shields D Fisher R Bayir H. Anti-Ferroptosis Drug Enhances Total-Body Irradiation Mitigation by Drugs That Block Apoptosis and Necroptosis. Radiat Res (2020) 193(5):435–50. doi: 10.1667/RR15486.1 PMC7299160 32134361

[208] Zhang X Tian M Li X Zheng C Wang A Feng J. Hematopoietic Protection and Mechanisms of Ferrostatin-1 on Hematopoietic Acute Radiation Syndrome of Mice. Int J Radiat Biol (2021) 97(4):464–73. doi: 10.1080/09553002.2021.1876956 33464146

[209] Li X Duan L Yuan S Zhuang X Qiao T He J. Ferroptosis Inhibitor Alleviates Radiation-Induced Lung Fibrosis (RILF) *via* Down-Regulation of TGF-β1. J Inflamm (Lond) (2019) 16:11. doi: 10.1186/s12950-019-0216-0 31160885 PMC6542066

[210] Lee H Zandkarimi F Zhang Y Meena JK Kim J Zhuang L. Energy-Stress-Mediated AMPK Activation Inhibits Ferroptosis. Nat Cell Biol (2020) 22(2):225–34. doi: 10.1038/s41556-020-0461-8 PMC7008777 32029897

[211] Li X Zhuang X Qiao T. Role of Ferroptosis in the Process of Acute Radiation-Induced Lung Injury in Mice. Biochem Biophys Res Commun (2019) 519(2):240–5. doi: 10.1016/j.bbrc.2019.08.165 31493867

[212] Bai Y-T Chang R Wang H Xiao F-J Ge R-L Wang L-S. ENPP2 Protects Cardiomyocytes From Erastin-Induced Ferroptosis. Biochem Biophys Res Commun (2018) 499(1):44–51. doi: 10.1016/j.bbrc.2018.03.113 29551679

[213] Tarangelo A Magtanong L Bieging-Rolett KT Li Y Ye J Attardi LD. P53 Suppresses Metabolic Stress-Induced Ferroptosis in Cancer Cells. Cell Rep (2018) 22(3):569–75. doi: 10.1016/j.celrep.2017.12.077 PMC5791910 29346757

[214] Wenzel SE Tyurina YY Zhao J St Croix CM Dar HH Mao G. PEBP1 Wardens Ferroptosis by Enabling Lipoxygenase Generation of Lipid Death Signals. Cell (2017) 171(3):628–41.e26. doi: 10.1016/j.cell.2017.09.044 29053969 PMC5683852

[215] Sun W-Y Tyurin VA Mikulska-Ruminska K Shrivastava IH Anthonymuthu TS Zhai Y-J. Phospholipase Ipla2β Averts Ferroptosis by Eliminating a Redox Lipid Death Signal. Nat Chem Biol (2021) 17(4):465–76. doi: 10.1038/s41589-020-00734-x PMC8152680 33542532

[216] Baba Y Higa JK Shimada BK Horiuchi KM Suhara T Kobayashi M. Protective Effects of the Mechanistic Target of Rapamycin Against Excess Iron and Ferroptosis in Cardiomyocytes. Am J Physiol Heart Circ Physiol (2018) 314(3):H659–68. doi: 10.1152/ajpheart.00452.2017 PMC5899260 29127238

[217] Chen D Fan Z Rauh M Buchfelder M Eyupoglu IY Savaskan N. ATF4 Promotes Angiogenesis and Neuronal Cell Death and Confers Ferroptosis in a xCT-Dependent Manner. Oncogene (2017) 36(40):5593–608. doi: 10.1038/onc.2017.146 PMC5633655 28553953

[218] Clemente LP Rabenau M Tang S Stanka J Cors E Stroh J. Dynasore Blocks Ferroptosis Through Combined Modulation of Iron Uptake and Inhibition of Mitochondrial Respiration. Cells (2020) 9(10):2259. doi: 10.3390/cells9102259 PMC7650611 33050207

[219] Doll S Freitas FP Shah R Aldrovandi M Da Silva MC Ingold I. FSP1 is a Glutathione-Independent Ferroptosis Suppressor. Nature (2019) 575(7784):693–8. doi: 10.1038/s41586-019-1707-0 31634899

[220] Doll S Proneth B Tyurina YY Panzilius E Kobayashi S Ingold I. ACSL4 Dictates Ferroptosis Sensitivity by Shaping Cellular Lipid Composition. Nat Chem Biol (2017) 13(1):91–8. doi: 10.1038/nchembio.2239 PMC5610546 27842070

[221] Fan B-Y Pang Y-L Li W-X Zhao C-X Zhang Y Wang X. Liproxstatin-1 is an Effective Inhibitor of Oligodendrocyte Ferroptosis Induced by Inhibition of Glutathione Peroxidase 4. Neural Regener Res (2021) 16(3):561–6. doi: 10.4103/1673-5374.293157 PMC7996026 32985488

[222] Liu P Feng Y Li H Chen X Wang G Xu S. Ferrostatin-1 Alleviates Lipopolysaccharide-Induced Acute Lung Injury via Inhibiting Ferroptosis. Cell Mol Biol Lett (2020) 25:10. doi: 10.1186/s11658-020-00205-0 32161620 PMC7045739

[223] NaveenKumar SK Hemshekhar M Kemparaju K Girish KS. Hemin-Induced Platelet Activation and Ferroptosis is Mediated Through ROS-Driven Proteasomal Activity and Inflammasome Activation: Protection by Melatonin. Biochim Biophys Acta Mol Basis Dis (2019) 1865(9):2303–16. doi: 10.1016/j.bbadis.2019.05.009 31102787

[224] Seibt TM Proneth B Conrad M. Role of GPX4 in Ferroptosis and its Pharmacological Implication. Free Radic Biol Med (2019) 133:144–52. doi: 10.1016/j.freeradbiomed.2018.09.014 30219704

[225] Villalpando-Rodriguez GE Blankstein AR Konzelman C Gibson SB. Lysosomal Destabilizing Drug Siramesine and the Dual Tyrosine Kinase Inhibitor Lapatinib Induce a Synergistic Ferroptosis Through Reduced Heme Oxygenase-1 (HO-1) Levels. Oxid Med Cell Longev (2019) 2019:9561281. doi: 10.1155/2019/9561281 31636810 PMC6766165

[226] Wang C Yuan W Hu A Lin J Xia Z Yang CF. Dexmedetomidine Alleviated Sepsis−Induced Myocardial Ferroptosis and Septic Heart Injury. Mol Med Rep (2020) 22(1):175–84. doi: 10.3892/mmr.2020.11114 PMC7248514 32377745

[227] Wang X Liu K Gong H Li D Chu W Zhao D. Death by Histone Deacetylase Inhibitor Quisinostat in Tongue Squamous Cell Carcinoma via Apoptosis, Pyroptosis, and Ferroptosis. Toxicol Appl Pharmacol (2021) 410:115363. doi: 10.1016/j.taap.2020.115363 33290780

[228] Yusuf RZ Saez B Sharda A van Gastel N Yu VWC Baryawno N. Aldehyde Dehydrogenase 3a2 Protects AML Cells From Oxidative Death and the Synthetic Lethality of Ferroptosis Inducers. Blood (2020) 136(11):1303–16. doi: 10.1182/blood.2019001808 PMC7483435 32458004

[229] Zhang Y Sun C Zhao C Hao J Zhang Y Fan B. Ferroptosis Inhibitor SRS 16-86 Attenuates Ferroptosis and Promotes Functional Recovery in Contusion Spinal Cord Injury. Brain Res (2019) 1706:48–57. doi: 10.1016/j.brainres.2018.10.023 30352209

[230] Ye LF Chaudhary KR Zandkarimi F Harken AD Kinslow CJ Upadhyayula PS. Radiation-Induced Lipid Peroxidation Triggers Ferroptosis and Synergizes With Ferroptosis Inducers. ACS Chem Biol (2020) 15(2):469–84. doi: 10.1021/acschembio.9b00939 PMC7180072 31899616

[231] Bi J Yang S Li L Dai Q Borcherding N Wagner BA. Metadherin Enhances Vulnerability of Cancer Cells to Ferroptosis. Cell Death Dis (2019) 10(10):682. doi: 10.1038/s41419-019-1897-2 31527591 PMC6746770

[232] Lei G Zhang Y Hong T Zhang X Liu X Mao C. Ferroptosis as a Mechanism to Mediate P53 Function in Tumor Radiosensitivity. Oncogene (2021) 40(20):3533–47. doi: 10.1038/s41388-021-01790-w PMC8141034 33927351

[233] Pan X Lin Z Jiang D Yu Y Yang D Zhou H. Erastin Decreases Radioresistance of NSCLC Cells Partially by Inducing GPX4-Mediated Ferroptosis. Oncol Lett (2019) 17(3):3001–8. doi: 10.3892/ol.2019.9888 PMC6365906 30854078

[234] Yuan Y Cao W Zhou H Qian H Wang H. CLTRN, Regulated by NRF1/RAN/DLD Protein Complex, Enhances Radiation Sensitivity of Hepatocellular Carcinoma Cells Through Ferroptosis Pathway. Int J Radiat Oncol Biol Phys (2021) 110(3):859–71. doi: 10.1016/j.ijrobp.2020.12.062 33508374

[235] Basit F van Oppen LM Schöckel L Bossenbroek HM van Emst-de Vries SE Hermeling JC. Mitochondrial Complex I Inhibition Triggers a Mitophagy-Dependent ROS Increase Leading to Necroptosis and Ferroptosis in Melanoma Cells. Cell Death Dis (2017) 8(3):e2716. doi: 10.1038/cddis.2017.133 28358377 PMC5386536

[236] Bersuker K Hendricks JM Li Z Magtanong L Ford B Tang PH. The CoQ Oxidoreductase FSP1 Acts Parallel to GPX4 to Inhibit Ferroptosis. Nature (2019) 575(7784):688–92. doi: 10.1038/s41586-019-1705-2 PMC6883167 31634900

[237] Chen P Li X Zhang R Liu S Xiang Y Zhang M. Combinative Treatment of β-Elemene and Cetuximab is Sensitive to KRAS Mutant Colorectal Cancer Cells by Inducing Ferroptosis and Inhibiting Epithelial-Mesenchymal Transformation. Theranostics (2020) 10(11):5107–19. doi: 10.7150/thno.44705 PMC7163451 32308771

[238] Dixon SJ Patel DN Welsch M Skouta R Lee ED Hayano M. Pharmacological Inhibition of Cystine-Glutamate Exchange Induces Endoplasmic Reticulum Stress and Ferroptosis. Elife (2014) 3:e02523. doi: 10.7554/eLife.02523 24844246 PMC4054777

[239] Kagan VE Mao G Qu F Angeli JPF Doll S Croix CS. Oxidized Arachidonic and Adrenic PEs Navigate Cells to Ferroptosis. Nat Chem Biol (2017) 13(1):81–90. doi: 10.1038/nchembio.2238 27842066 PMC5506843

[240] Lin P-L Tang H-H Wu S-Y Shaw N-S Su C-L. Saponin Formosanin C-Induced Ferritinophagy and Ferroptosis in Human Hepatocellular Carcinoma Cells. Antioxid (Basel) (2020) 9(8):682. doi: 10.3390/antiox9080682 PMC7463707 32751249

[241] Ma S Henson ES Chen Y Gibson SB. Ferroptosis is Induced Following Siramesine and Lapatinib Treatment of Breast Cancer Cells. Cell Death Dis (2016) 7:e2307. doi: 10.1038/cddis.2016.208 27441659 PMC4973350

[242] Llabani E Hicklin RW Lee HY Motika SE Crawford LA Weerapana E. Diverse Compounds From Pleuromutilin Lead to a Thioredoxin Inhibitor and Inducer of Ferroptosis. Nat Chem (2019) 11(6):521–32. doi: 10.1038/s41557-019-0261-6 PMC6639018 31086302

[243] Shin D Kim EH Lee J Roh J-L. Nrf2 Inhibition Reverses Resistance to GPX4 Inhibitor-Induced Ferroptosis in Head and Neck Cancer. Free Radic Biol Med (2018) 129:454–62. doi: 10.1016/j.freeradbiomed.2018.10.426 30339884

[244] Venkatesh D O’Brien NA Zandkarimi F Tong DR Stokes ME Dunn DE. MDM2 and MDMX Promote Ferroptosis by Pparα-Mediated Lipid Remodeling. Genes Dev (2020) 34(7-8):526–43. doi: 10.1101/gad.334219.119 PMC7111265 32079652

[245] Wang Y Tang M. PM2.5 Induces Ferroptosis in Human Endothelial Cells Through Iron Overload and Redox Imbalance. Environ Pollut (2019) 254(Pt A):112937. doi: 10.1016/j.envpol.2019.07.105 31401526

[246] Wei S Qiu T Yao X Wang N Jiang L Jia X. Arsenic Induces Pancreatic Dysfunction and Ferroptosis via Mitochondrial ROS-Autophagy-Lysosomal Pathway. J Hazard Mater (2020) 384:121390. doi: 10.1016/j.jhazmat.2019.121390 31735470

[247] Zhang Y Tan H Daniels JD Zandkarimi F Liu H Brown LM. Imidazole Ketone Erastin Induces Ferroptosis and Slows Tumor Growth in a Mouse Lymphoma Model. Cell Chem Biol (2019) 26(5):623–33.e9. doi: 10.1016/j.chembiol.2019.01.008 30799221 PMC6525071

[248] Wang L Liu Y Du T Yang H Lei L Guo M. ATF3 Promotes Erastin-Induced Ferroptosis by Suppressing System Xc. Cell Death Differ (2020) 27(2):662–75. doi: 10.1038/s41418-019-0380-z PMC7206049 31273299

[249] Fukumoto R Cary LH Gorbunov NV Lombardini ED Elliott TB Kiang JG. Ciprofloxacin Modulates Cytokine/Chemokine Profile in Serum, Improves Bone Marrow Repopulation, and Limits Apoptosis and Autophagy in Ileum After Whole Body Ionizing Irradiation Combined With Skin-Wound Trauma. PloS One (2013) 8(3):e58389. doi: 10.1371/journal.pone.0058389 23520506 PMC3592826

[250] Kim J-S Yang M Lee C-G Kim S-D Kim J-K Yang K. *In Vitro* and *In Vivo* Protective Effects of Granulocyte Colony-Stimulating Factor Against Radiation-Induced Intestinal Injury. Arch Pharm Res (2013) 36(10):1252–61. doi: 10.1007/s12272-013-0164-9 23728838

[251] Su L Wang Z Huang F Lan R Chen X Han D. 18β-Glycyrrhetinic Acid Mitigates Radiation-Induced Skin Damage via NADPH Oxidase/ROS/p38MAPK and NF-κb Pathways. Environ Toxicol Pharmacol (2018) 60:82–90. doi: 10.1016/j.etap.2018.04.012 29677640

[252] Mirzoeva S Paunesku T Wanzer MB Shirvan A Kaempfer R Woloschak GE. Single Administration of P2ta (AB103), a CD28 Antagonist Peptide, Prevents Inflammatory and Thrombotic Reactions and Protects Against Gastrointestinal Injury in Total-Body Irradiated Mice. PloS One (2014) 9(7):e101161. doi: 10.1371/journal.pone.0101161 25054224 PMC4108308

[253] Li XH Ghosh SP Ha CT Fu D Elliott TB Bolduc DL. Delta-Tocotrienol Protects Mice From Radiation-Induced Gastrointestinal Injury. Radiat Res (2013) 180(6):649–57. doi: 10.1667/RR13398.1 24294967

[254] Wang J Zheng J Kulkarni A Wang W Garg S Prather PL. Palmitoylethanolamide Regulates Development of Intestinal Radiation Injury in a Mast Cell-Dependent Manner. Dig Dis Sci (2014) 59(11):2693–703. doi: 10.1007/s10620-014-3212-5 PMC4213290 24848354

[255] Peng Z Xu Z Wen W Wang R. Tea Polyphenols Protect Against Irradiation-Induced Injury in Submandibular Glands’ Cells: A Preliminary Study. Arch Oral Biol (2011) 56(8):738–43. doi: 10.1016/j.archoralbio.2010.12.009 21292239

[256] Zhang H Yan H Zhou X Wang H Yang Y Zhang J. The Protective Effects of Resveratrol Against Radiation-Induced Intestinal Injury. BMC Complement Altern Med (2017) 17(1):410. doi: 10.1186/s12906-017-1915-9 28814292 PMC5559783

[257] Pratheeshkumar P Kuttan G. Protective Role of Vernonia Cinerea L. Against Gamma Radiation–Induced Immunosupression and Oxidative Stress in Mice. Hum Exp Toxicol (2011) 30(8):1022–38. doi: 10.1177/0960327110385959 20930026

[258] Sezer A Usta U Kocak Z Yagci MA. The Effect of a Flavonoid Fractions Diosmin + Hesperidin on Radiation-Induced Acute Proctitis in a Rat Model. J Cancer Res Ther (2011) 7(2):152–6. doi: 10.4103/0973-1482.82927 21768702

[259] Kalita B Ranjan R Singh A Yashavarddhan MH Bajaj S Gupta ML. A Combination of Podophyllotoxin and Rutin Attenuates Radiation Induced Gastrointestinal Injury by Negatively Regulating NF-κb/P53 Signaling in Lethally Irradiated Mice. PloS One (2016) 11(12):e0168525. doi: 10.1371/journal.pone.0168525 28036347 PMC5201299

[260] Lu J Wang C Yang M Zhao H Liu Y Cao X. Effect of Modified Zhuye Shigao Decoction and its Components on Preventing Radiation Esophagitis of Rats. Chin J Integr Med (2014) 20(6):462–7. doi: 10.1007/s11655-014-1754-1 24952170

[261] Sha H Gu Y Shen W Zhang L Qian F Zhao Y. Rheinic Acid Ameliorates Radiation-Induced Acute Enteritis in Rats Through PPAR-γ/NF-κb. Genes Genomics (2019) 41(8):909–17. doi: 10.1007/s13258-019-00824-8 31037524

[262] Khayyal MT Kreuter MH Kemmler M Altmann P Abdel-Naby DH El-Ghazaly MA. Effect of a Chamomile Extract in Protecting Against Radiation-Induced Intestinal Mucositis. Phytother Res (2019) 33(3):728–36. doi: 10.1002/ptr.6263 30632234

[263] Takahashi A Inoue H Mishima K Ide F Nakayama R Hasaka A. Evaluation of the Effects of Quercetin on Damaged Salivary Secretion. PloS One (2015) 10(1):e0116008. doi: 10.1371/journal.pone.0116008 25629520 PMC4309588

[264] Ito I Loucas BD Suzuki S Kobayashi M Suzuki F. Glycyrrhizin Protects γ-Irradiated Mice From Gut Bacteria-Associated Infectious Complications by Improving miR-222-Associated Gas5 RNA Reduction in Macrophages of the Bacterial Translocation Site. J Immunol (2020) 204(5):1255–62. doi: 10.4049/jimmunol.1900949 PMC7033010 31941655

[265] Sharma D Goel HC Chauhan S. Radioprotective Potential of Lagenaria Siceraria Extract Against Radiation-Induced Gastrointestinal Injury. Appl Physiol Nutr Metab (2016) 41(12):1248–54. doi: 10.1139/apnm-2016-0136 27863208

[266] Yoon WS Kim CY Yang DS Park YJ Park W Ahn YC. Protective Effect of Triphala on Radiation Induced Acute Intestinal Mucosal Damage in Sprague Dawley Rats. Indian J Exp Biol (2012) 50(3):195–200. 22439434

[267] Radwan RR Karam HM. Resveratrol Attenuates Intestinal Injury in Irradiated Rats *via* PI3K/Akt/mTOR Signaling Pathway. Environ Toxicol (2020) 35(2):223–30. doi: 10.1002/tox.22859 31633274

[268] Kratochwil C Flechsig P Lindner T Abderrahim L Altmann A Mier W. 68Ga-FAPI PET/CT: Tracer Uptake in 28 Different Kinds of Cancer. J Nucl Med (2019) 60(6):801–5. doi: 10.2967/jnumed.119.227967 PMC6581228 30954939

[269] Poty S Mandleywala K O’Neill E Knight JC Cornelissen B Lewis JS. 89Zr-PET Imaging of DNA Double-Strand Breaks for the Early Monitoring of Response Following α- and β-Particle Radioimmunotherapy in a Mouse Model of Pancreatic Ductal Adenocarcinoma. Theranostics (2020) 10(13):5802–14. doi: 10.7150/thno.44772 PMC7255009 32483420

[270] Kantara C Moya SM Houchen CW Umar S Ullrich RL Singh P. Novel Regenerative Peptide TP508 Mitigates Radiation-Induced Gastrointestinal Damage by Activating Stem Cells and Preserving Crypt Integrity. Lab Invest (2015) 95(11):1222–33. doi: 10.1038/labinvest.2015.103 PMC4626368 26280221

